# Structural insights into molecular mechanism for *N*^6^-adenosine methylation by MT-A70 family methyltransferase METTL4

**DOI:** 10.1038/s41467-022-33277-x

**Published:** 2022-09-26

**Authors:** Qiang Luo, Jiezhen Mo, Hao Chen, Zetao Hu, Baihui Wang, Jiabing Wu, Ziyu Liang, Wenhao Xie, Kangxi Du, Maolin Peng, Yingping Li, Tianyang Li, Yangyi Zhang, Xiaoyan Shi, Wen-Hui Shen, Yang Shi, Aiwu Dong, Hailin Wang, Jinbiao Ma

**Affiliations:** 1 State Key Laboratory of Genetic Engineering, Collaborative Innovation Center of Genetics and Development, Department of Biochemistry and Biophysics, Institute of Plant Biology, School of Life Sciences, Fudan University, 200438 Shanghai, China; 2 State Key Laboratory of Genetic Engineering, Collaborative Innovation Center of Genetics and Development, International Associated Laboratory of CNRS-Fudan-HUNAU on Plant Epigenome Research, Department of Biochemistry and Biophysics, Institute of Plant Biology, School of Life Sciences, Fudan University, 200438 Shanghai, China; 3 State Key Laboratory of Environmental Chemistry and Ecotoxicology, Research Center for Eco-Environmental Sciences, Chinese Academy of Sciences, University of Chinese Academy of Sciences, 100085 Beijing, China; 4 Department of Human Cell Biology and Genetics, School of Medicine, Southern University of Science and Technology, 518055 Shenzhen, Guangdong China; 5 Institut de Biologie Moléculaire des Plantes, CNRS, Université de Strasbourg, 12 rue du Général Zimmer, 67084 Strasbourg Cédex, France; 6 Ludwig Institute for Cancer Research, Oxford University, Old Road Campus Research Building, Roosevelt Dr, Headington, Oxford, OX3 7DQ UK; 7 Institute of Environment and Health, Jianghan University, 430056 Wuhan, Hubei China

**Keywords:** X-ray crystallography, RNA modification

## Abstract

METTL4 belongs to a subclade of MT-A70 family members of methyltransferase (MTase) proteins shown to mediate *N*^6^-adenosine methylation for both RNA and DNA in diverse eukaryotes. Here, we report that Arabidopsis METTL4 functions as U2 snRNA MTase for *N*^6^−2’-O-dimethyladenosine (m^6^Am) in vivo that regulates flowering time, and specifically catalyzes *N*^6^-methylation of 2’-O-methyladenosine (Am) within a single-stranded RNA in vitro. The apo structures of full-length Arabidopsis METTL4 bound to S-adenosyl-L-methionine (SAM) and the complex structure with an Am-containing RNA substrate, combined with mutagenesis and in vitro enzymatic assays, uncover a preformed L-shaped, positively-charged cavity surrounded by four loops for substrate binding and a catalytic center composed of conserved residues for specific Am nucleotide recognition and *N*^6^-methylation activity. Structural comparison of METTL4 with the mRNA m6A enzyme METTL3/METTL14 heterodimer and modeling analysis suggest a catalytic mechanism for *N*^6^-adenosine methylation by METTL4, which may be shared among MT-A70 family members.

## Abstract (web summary)

Here the authors combine functional and structural work to show that METTL4 functions as U2 snRNA m6Am RNA methyltransferase supporting N6-methylation of 2’-O-methyladenosine in Arabidopsis.

## Introduction

Enzymatic RNA covalent modifications represent an essential epigenetic mechanism that exquisitely and plastically regulates multiple cellular activities in eukaryotes^1^. One of the most abundant modifications is the methylation decorated on the *N*^6^ of adenosine (m^6^A), which is widespread in mRNA^2,3^, small nuclear RNA (snRNA)^4^, long noncoding RNA (lncRNA)^5,6^, and ribosomal RNA (rRNA)^7^. The m^6^A decoration of these RNA molecules regulates RNA structure or modulates protein-RNA interaction^8,9^, impacting RNA metabolism and diverse signaling pathways essential for cell survival and differentiation^10,11^. Abnormal m^6^A-based RNA metabolism has been suggested to cause human diseases including obesity^12^ and cancers^13,14^. Recent studies have identified the enzymes that mediate m^6^A modification on diverse RNAs, including the METTL3/METTL14 heterodimer^15^, METTL16^16^, ZCCHC4^17^, and METTL5^18^ with exquisite sequence specificity. METTL3/METTL14 primarily catalyzes the modification of mRNA and lncRNA nearby the stop code and in the 3′UTR by recognizing a DRACH (D: A, G, U; R: G, A; H: A, C, U) consensus sequence^19–21^. Some mRNAs and snRNAs with an UACAGAGAA motif in the stem-loop region are decorated by METTL16^16^.

METTL4, which belongs to a subclade of the MT-A70 family of proteins but being separated from the METTL3 and METTL14 subclades^22^, has been reported to be a U2 snRNA *N*^6^-adenosine methyltransferase in human and Drosophila^23–25^ Of note, while human METTL4 only methylates U2 snRNA on A30 when the 2′-OH on the ribose ring is methylated^23,24^, Drosophila METTL4 does not have this requisite and can methylate the same adenosine in U2 snRNA without 2′-O methylation^25^. Interestingly, METTL4 or its homologs have also been shown to deposit adenine *N*^6^ methylation in DNA (^6^mA) in the genome of mammalian tissues^26–28^ or mediate DNA ^6^mA methylation in mitochondria^29^. However, the exact substrate of METTL4 is still under debate in part because of the lack of structural evidence for any proposed METTL4 enzymatic activities discussed above. In fact, only apo structures of MT-A70 family homolog proteins, METTL3/METTL14 heterodimer in complex with cofactors were reported previously^15,30,31^.

Here we show that Arabidopsis METTL4 functions as U2 snRNA MTase for *N*^6^−2′-O-dimethyladenosine (m^6^Am) in vivo, and specifically catalyzes *N*^6^-methylation of 2′-O-methyladenosine (Am) within a single-stranded RNA in vitro. We determined crystal structures of full-length METTL4 protein and its complexes with S-adenosyl-L-methionine (SAM) or its analogs, and co-crystal structure of METTL4 bound an Am-containing RNA substrate in the presence of S-adenosyl-L-homocysteine (SAH). In combination with biochemical and modeling analysis, our findings shed light on the specificity of Am recognition by METTL4 and a novel molecular mechanism for *N*^6^-adenosine methylation, which is likely to be shared by MT-A70 family MTases, such as METTL3/METTL14.

## Results

### In vivo functions of METTL4 in Arabidopsis

**Fig. 1:**
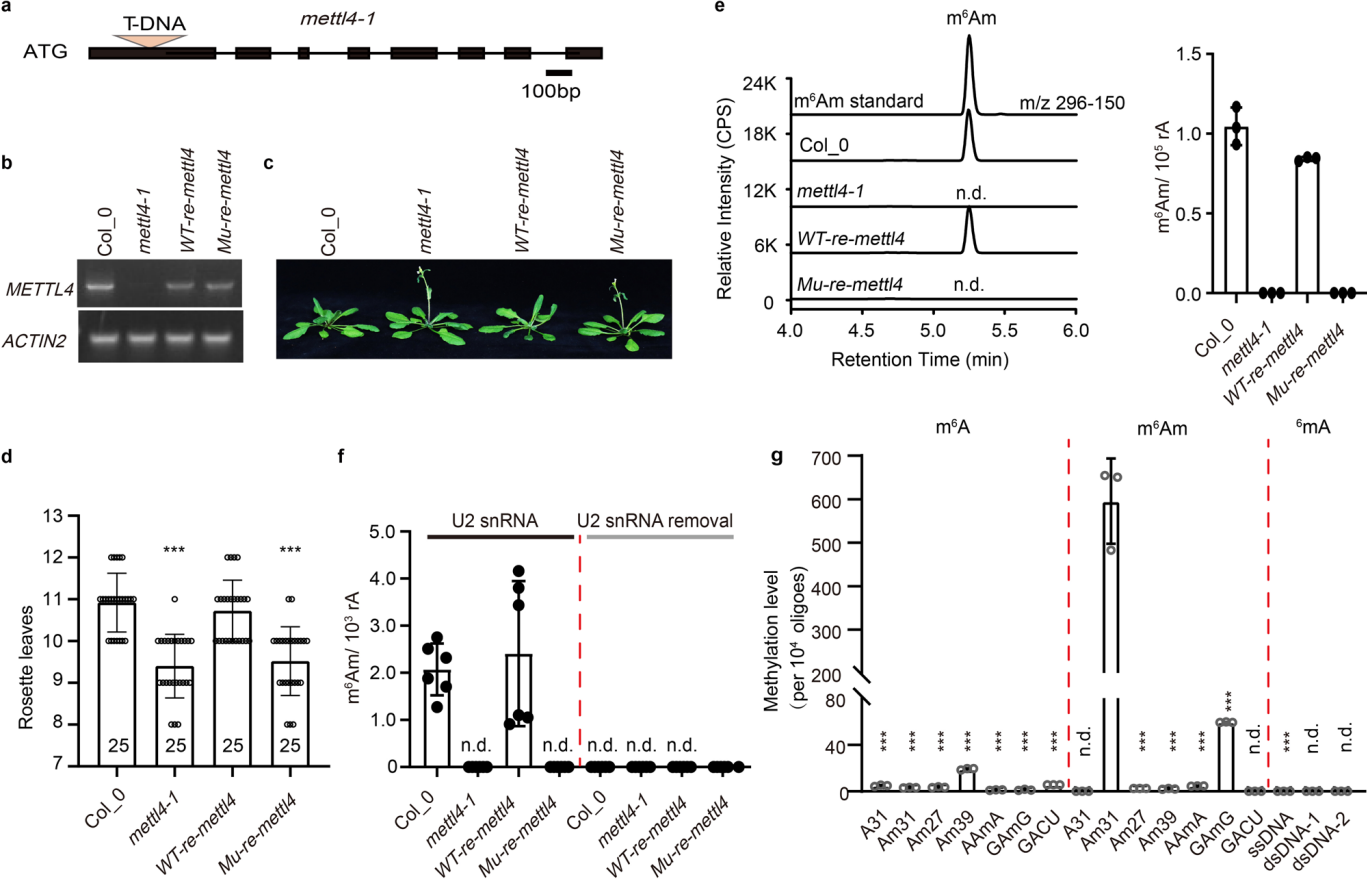
METTL4 mediates solely m^6^Am methylation of U2 snRNA in *Arabidopsis thaliana*. **a** The diagrammatic sketch of the T-DNA insertion site in the *METTL4* gene. **b** RT-PCR validation of the full-length transcript of the *METTL4* gene, the *AtACTIN2* transcript acts as a control. **c** The phenotype of the Arabidopsis plants grown in long-day (LD) condition (22 °C and 16-h-light/8-h-dark). **d** Statistics of the number of rosette leaves when flower bunds first appeared, which correlates with flowering time. And the number of the calculated plants were shown in the bar column. The APPA mutant was generated from conserved DPPW motif. Wild-type *METTL4* and *APPA* mutant gene rescued *mettl4-1* plants are shown as *WT-re-mettl4* and *Mu-re-mettl4*, respectively. ***Significant differences between WT and mutant (Student’s *t* test: ***P < 0.001). **e** UPLC-MS/MS chromatograms (left) and quantification of m^6^Am abundance in total RNA from *Arabidopsis thaliana* (right). The error bars represent standard deviations. **f** The quantification of m^6^Am abundance in U2 snRNA and residual RNAs. **g** Enzymatic assay of METTL4 using synthetic U2 snRNA fragments and various RNA and DNA oligoes as substrates in vitro. The error bars represent standard deviations. n.d., not detectable; *p* < 0.001.

Based on phylogenetic analysis^32^, we identified At1G19340 in *Arabidopsis thaliana* as the mammalian METTL4 ortholog and systematically investigated its potential substrates in vitro and in vivo. We firstly confirmed a T-DNA insertion mutant line, *mettl4-1*, in which the T-DNA was inserted into the first exon of the *METTL4* gene thus resulting in the disruption of the full-length *METTL4* transcript (Fig. 1a, b). This *mettl4-1* mutant displayed an early flowering phenotype under long-day (LD) conditions compared with the wild-type Col-0 (Fig. 1c). The early flowering phenotype of *mettl4-1* mutant was rescued by re-introduction of a wild type copy of the *METTL4* gene but not a catalytic inactive mutant, in which the conserved DPPW motif critical for catalysis was mutated to APPA (Fig. 1c, d), indicating that the developing defect of *mettl4-1* early flowering phenotype is due to the loss of the catalytic activity of METTL4. Next, we investigated the potential substrates of Arabidopsis METTL4 using ultra-high-performance liquid chromatography-triple quadrupole mass spectrometry, coupled with multiple-reaction monitoring (UHPLC-MRM-MS/MS). We measured four RNA modifications, including *N*^1^-methyladenosine (m^1^A), 2′-O-methyladenosine (Am), *N*^6^-methyladenosine (m^6^A), and *N*^6^−2′-O-dimethyladenosine (m^6^Am), in total RNA extracted from Col-0, *mettl4-1* mutant and the two rescued plants (Supplementary Fig. 1a–f and Fig. 1e). The quantification of m^1^A, Am and m^6^A showed similar levels in wild-type and *mettl4-1* mutant plants, suggesting that these modifications were not affected by the loss of METTL4. However, the m^6^Am level was significantly compromised in the *mettl4-1* mutant plant but restored by the reintroduction of a wild-type copy of *METTL4*. Importantly, the m^6^Am level of U2 snRNA was barely detectable in the *mettl4-1* mutant or in the catalytic mutant *METTL4 APPA* rescued plant, but the level was restored in the wild-type *METTL4* rescued plant (Fig. 1f). Moreover, when the RNA extracts were deprived of U2 snRNA, no m^6^Am was detected (Fig. 1f), suggesting that METTL4-dependent internal m^6^Am modification is likely only present in U2 snRNA in Arabidopsis. Taken together, these results suggested that METTL4 is the sole m^6^Am RNA methyltransferase in Arabidopsis and is exclusively responsible for U2 snRNA m^6^Am modification in vivo.

To address why depletion of *METTL4* (Supplementary Fig. 2a) resulted in an early flowering phenotype (Fig. 1c), we performed RNA sequencing to investigate the molecular function of *METTL4* in Arabidopsis (Supplementary Fig. 2a). Compared with the wild-type Col-0, there are 127 up-regulated genes and 119 down-regulated genes in the *mettl4* mutant, respectively (Supplementary Fig. 2b). Gene Ontology (GO) analysis showed that a set of genes involved in photosynthesis and response to cold or freezing are among these 127 up-regulated and 119 down-regulated genes, which are related to flowering time regulation (Supplementary Fig. 2c). Interestingly, many genes (497) displayed splicing changes in the *mettl4* mutant compared to the wild-type Col-0 (Supplementary Fig. 2d), which are mainly involved in the basic RNA biosynthetic and metabolic processes (Supplementary Fig. 2e). Although there is no direct association between early flowering phenotype and the genes with obvious splicing alterations, it is plausible that Arabidopsis METTL4 affected pre-messenger RNA (pre-mRNA) splicing of a set of these genes, similar to the function of mammalian METTL4^23,24^.

### Substrate features of the Arabidopsis METTL4 in vitro

To further clarify the substrate specificities of the Arabidopsis METTL4, we performed in vitro enzymatic activity analysis using full-length recombinant METTL4 protein purified from *E. coli*. Unlike the human U2 snRNA, which has only one Am decorated site (Am30), the Arabidopsis U2 snRNA contains two Am sites (Am31 and Am39)^33^. Therefore, we designed three RNA substrates based on the Arabidopsis U2 snRNA sequences: Am31 for 2′-OH and 2′-OMe at site 31, Am39 for 2′-OMe at site 39, and Am27 for 2′-OMe at site 27 as a control. Interestingly, only Am31, but not Am39 and Am27, could be methylated by METTL4 in vitro (Fig. 1g and Supplementary Fig. 3a, b). Changing the sequence around the Am31 site from AAmG to GAmG reduced the in vitro activities of METTL4, and the enzyme activity is almost completely lost when the wild-type sequence was changed to AAmA (Fig. 1g and Supplementary Fig. 3a, b). These results suggest that sequences or secondary structures around the Am nucleotide may be critical for the enzyme activity. Next, we tested whether the 2′-O-methyl modification is necessary for the enzyme activity of METTL4 in vitro. METTL4 showed weak m^6^A enzyme activities towards probes Am39 and GACU with two 5′-GGACU-3′ motifs (optimal sequence for METTL3/METTL14 heterodimer^34^), and negligible activities towards most other probes, in comparison to the high m6Am enzyme activity to Probe Am31 (Fig. 1g and Supplementary Fig. 3a–c), indicating that METTL4 prefers Am over A in vitro. In addition, the quantification of m^6^A modification on U2 snRNA and total RNA showed similar levels in wild-type and *mettl4-1* mutant plants (Supplementary Fig. 1g), suggesting that METTL4 decorates only on Am31 in U2 snRNA but not on A or Am at other positions of U2 snRNA or other RNAs in vivo. These results strongly suggest that Arabidopsis METTL4 has stringent specificities for the sequences and secondary structures surrounding Am31 nucleotide in the Arabidopsis U2 snRNA. We also measured Arabidopsis METTL4 activities on ssDNA and dsDNA. Compared to the RNA substrates, only a negligible level of activity was observed when a single strand DNA (ssDNA) probe containing the identical sequence of Probe Am31 was used as a substrate, and no detectable DNA ^6^mA signal was observed on double-stranded DNA (dsDNA) substrates (Fig. 1g and Supplementary Fig. 3a, b). Taken together, these results suggest that Arabidopsis METTL4 prefers Am-containing RNA as a substrate in vitro.

### The crystal structure of the full-length METTL4

**Fig. 2:**
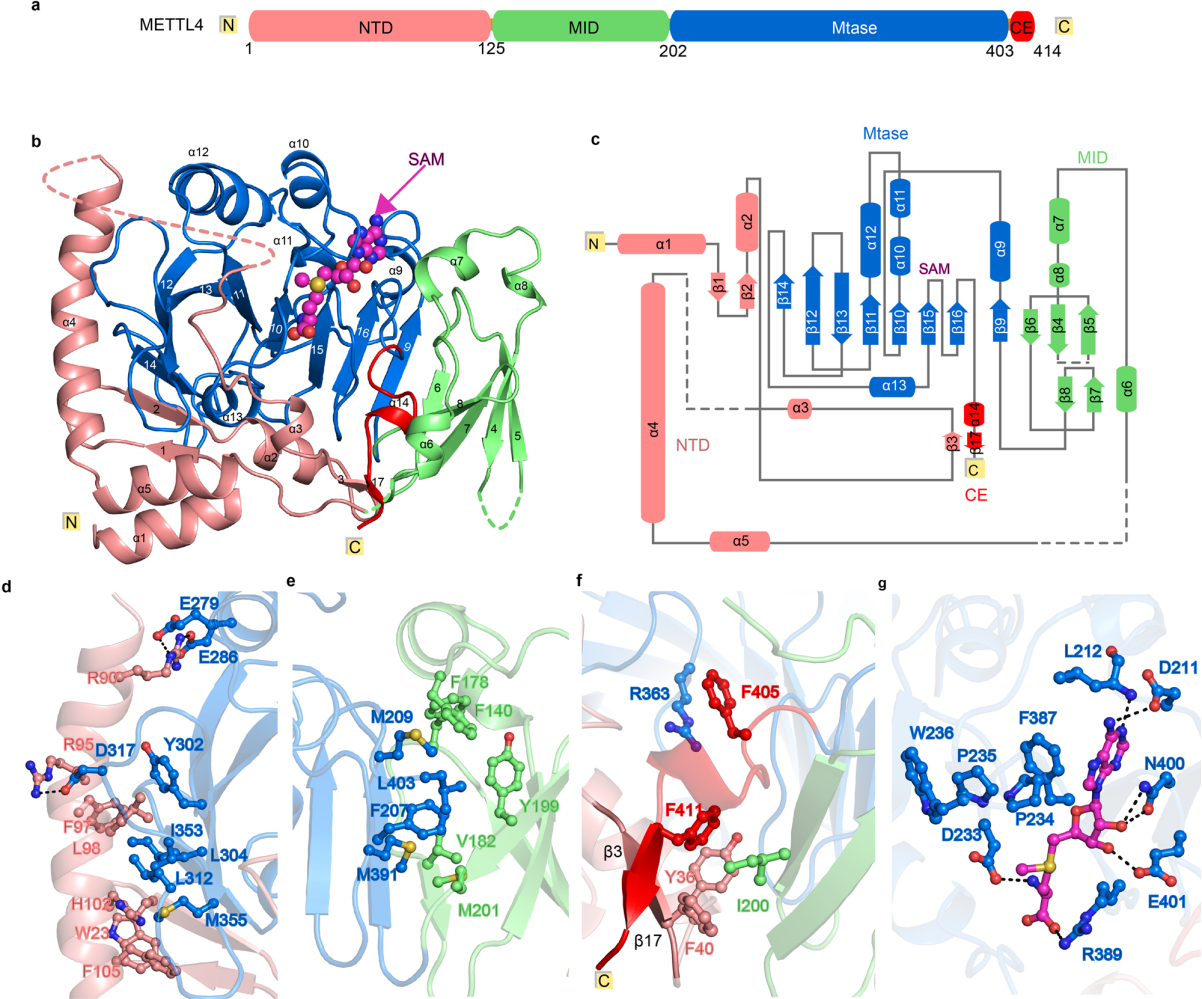
Crystal structure of full-length METTL4 bound to SAM. **a** Schematic domain structures of METTL4. **b** Cartoon representation of full-length METTL4 bound to SAM, with the β-strands and α-helices numbered numerically and the invisible residues in electric map shown as dashed lines. The SAM is shown as magenta sphere. **c** Schematic representation of the full-length structure of METTL4. **d**–**f** Close-up view of the interaction between MTase domain and NTD (**d**), MID (**e**), and CE domain (**f**). **g** The interaction network between METTL4 and SAM. The hydrogen bond interactions are shown as green dashed lines and the Vander Waa interactions are shown as red dashed fans. **f** Measurement of the binding affinity between SAM and the METTL4 wild-type and mutated proteins using ITC, n.d., not detected.

To elucidate the molecular mechanism of METTL4-mediated *N*^6^-adenosine methylation on U2 snRNA, we performed structural analysis by crystallization of the recombinant full-length protein. Sequence alignment suggested that Arabidopsis METTL4 contains a conserved MTase domain (residues 203-403) with a strictly conserved DPPW motif shared by the MT-A70 m^6^A methyltransferases, as well as an N-terminal extended region without known structural information (Supplementary Fig. 4). We solved the crystal structures of the full-length METTL4 alone (apo), as well as METTL4 bound to the methyl donor S-adenosyl-L-methionine (SAM), or its product S-adenosyl-L-homocysteine (SAH), or its analog Sinefungin (SFG) (Supplementary Fig. 5a–d and Tables 1 and  2). Due to the high structural similarity (Supplementary Figs. 5 and 6), we chose the structure of METTL4-SAM complex for subsequent analysis. Of note, the central MTase domain of METTL4 possessing a classic sandwich-fold similar to METTL3, is embraced by two domains from the N-terminal extended region, i.e., the N-terminal domain (NTD), and the middle domain (MID; Fig. 2a–c). NTD is mainly composed of five α-helixes (α 1–5) and three β-strands (β 1–3). A loop between α13 and β14 in MTase domain, referred to as Interface loop in METTL3, is stabilized by the long α4 of NTD via extensive hydrophobic interactions in combination with charged interactions (Fig. 2d), which is likely conserved based on sequence alignment (Supplementary Fig. 4). MID, mainly composed of five β-strands and three short α-helices with an Ig-like fold, leans to the side of the β9 strand of the MTase domain primarily via antiparallel β-sheet (β6↓-β9↑) and hydrophobic interactions (Fig. 2e). In addition, the C-terminal extension (CE, aa:404-414), stretching out from the Mtase domain, forms an antiparallel beta sheet with NTD β3 strand and hydrophobic interactions with I200 from the MID domain (Fig. 2f), which locks the NTD and MID domains together like a zip. The SAM molecule is bound adjacent to the conserved DPPW motif of Mtase domain in the complex structure (Fig. 2g and Supplementary Fig. 7), and particularly, aspartic acid D233 of DPPW motif forms a hydrogen bond with the amino group of SAM tail. Most of the residues involved in SAM binding in METTL4 are conserved in METTL3 (Supplementary Fig. 8), suggesting that MTase domains of MT-A70 family proteins share a common SAM binding mode. However, unlike METTL3, which requires METTL14 to form a fully functional enzyme, METTL4 alone forms an integrated structure by extensive interactions of the central MTase domain with NTD, MID and CE, to perform enzyme function.

**Table 1:** The data collection of SeMETTL4 for structure determination

	SeMETTL4-MAD		
Data collection			
Space group	P21	P21	P21
Cell parameter			
* a, b, c* (Å)	60.1, 94.2, 95.7	60.3, 94.4, 96.0	60.2, 94.3, 95.5
*α*, *β*, *γ* (°)	90.0, 101.8, 90.0	90.0, 101.8, 90.0	90.0, 101.8, 90.0
Wavelength(Å)	0.96108	0.97956	0.97943
Resolution (Å)	30.0-2.80	30.0-2.80	30.0-2.80
Last shell (Å)	(2.90-2.80)	(2.90-2.80)	(2.90-2.80)
Completeness (%)	99.3 (99.0)	99.6 (98.0)	99.2 (98.1)
Redundancy	1.9 (1.9)	3.7 (3.5)	1.9 (1.9)
I/σ(I)	9.9 (1.2)	10.5 (0.8)	9.9 (1.0)
* Rmerge* (%)	6.2 (50.8)	9.2 (108.8)	5.9 (57.3)
No. reflections	50,092 (5006)	26,052 (2544)	50,458 (4970)
CC^*^	0.844^a^	0.691^a^	0.811^a^

^a^The values in parentheses are for highest resolution shell.

^b^The statistics CC^b^ is an estimate of the “true” CC data under examination to the (unknown) true intensities.

**Table 2:** Data collection and refinement statistics

	SeMETTL4	METTL4- SAM	METTL4- SAH	METTL4- SFG	METTL4-Am-SAH
Space group	P21	P21	P21	P212121	I222
*a, b, c* (Å)	60.8,94.3, 95.7	61.9,93.7, 96.3	63.6,93.5, 96.6	52.2,84.6, 94.1	93.8,130.0, 141.3
α, β, γ (°)	90.0,101.7,90.0	90.0,104.3,90.0	90.0,104.7,90.0	90.0,90.0, 90.0	90.0,90.0, 90.0
Wavelength(Å)	0.97930	0.97916	0.97776	0.97853	0.97852
Resolution (Å)	30.0-2.50	30.0-2.30	30.0-2.45	30.0-2.35	30.0-3.0
Last shell (Å)	(2.59-2.50)^a^	(2.38-2.30)^a^	(2.54-2.45)^a^	(2.43-2.35)^a^	(3.11-3.00)^a^
Completeness (%)	98.2(93.7)	98.1(91.1)	97.6(88.6)	98.8(91.5)	82.4(54.7)
Redundancy	4.8(3.3)	3.4(3.1)	5.3(4.0)	11.8(8.6)	5.8(2.4)
I/σ(I)	29.9(7.2)	25.6(2.0)	17.5(2.4)	24.2(3.3)	7.8(1.9)
*Rmerge* (%)	13.2(41.3)	6.3(59.7)	10.4(39.2)	17.4(60.6)	11.2(40.0)
No. reflections	36127	46526	39010	17743	14468
	(3421)	(4278)	(3536)	(1596)	(938) ^a^
Refinement					
Resolution (Å)	2.50	2.30	2.45	2.35	3.01
R_work_ (%) / R_free_ (%)	22.6/26.5	22.0/25.1	21.1/25.4	20.1/23.7	24.0/30.0
No. of atoms					
Protein	5326	5515	5639	2741	2877
RNA/DNA	0	0	0	0	48
Ligand/ion	0	54	96	45	26
Water	11	57	22	61	19
* B* factors	50.0	72.0	55.0	47.0	94.0
Bond length (Å)	0.007	0.006	0.005	0.003	0.019
Bond angle (°)	1.010	1.080	0.974	0.072	1.982
Ramachandran plot (%)					
Most favored	96.31	97.41	98.11	5.38	89.82
Additional allowed	3.69	2.59	1.89	4.62	10.15
Outliers	0	0	0	0	0
PDB number	7CVA	7CV7	7CV9	7CV8	7CV6

Data are from one crystal for each structure.

^a^Values in parentheses are for highest-resolution shell.

### Complex structure of METTL4 bound to Am

**Fig. 3:**
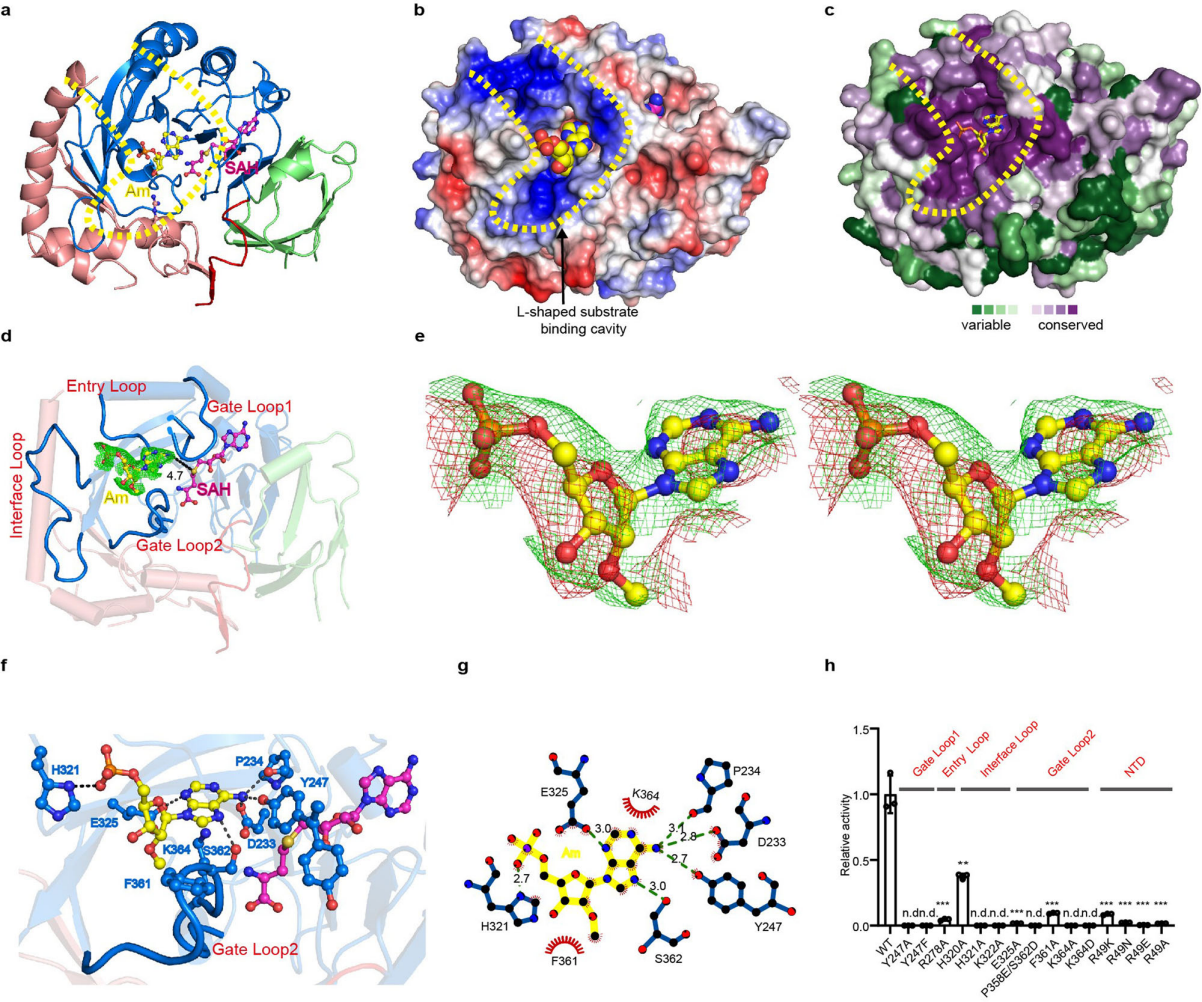
Crystal structure of the ternary complex of METTL4-Am-SAH uncovers a L-shaped cavity for the substrate binding and a conserved pocket for Am recognition. **a** Overall view of the METTL4-Am-SAH complex. **b** Electric surface of METTL4. The positive charged cleft is outlined by yellow dashed line, and the Am is shown as sphere. **c** ConSurf analysis of the conservation of METTL4 proteins. **d** Four conserved loops form substrate-binding cavity, and key residues involved in Am recognition are shown in stick and ball. The 2Fo-Fc map of Am is contoured at the 1.0 sigma level, and the invisible residues in electron density map of Gate Loop 1 shown as dashed lines. **e** Cross-eyed stereo view of the 2Fo-Fc map in green color at 1.0 sigma level generated by CCP4 program and Fo-Fc omit map in red color at 0.6 sigma level generated by Phenix program of the Am molecule in the catalytic pocket of METTL4. The estimated occupancy of the Am molecule in the catalytic pocket is 0.78 according to phenix.refine program. **f** Close-up view of the interactions between Am and MTase domain. The hydrogen bond interactions are depicted as yellow dashed lines. **g** The diagram of interactions involving in Am was analyzed by LigPlot. The hydrogen bonds are indicated by dashed lines and the hydrophobic contacts are shown as an arc with spokes radiating. **h** Enzymatic assay of METTL4 mutants measured by UHPLC-MRM-MS/MS method. The data are mean ± SD. Statistical analysis used Student’s test for the difference wild-type and mutants, n.s. not significant, n.d. not detectable; **p < 0.01; ***p < 0.001.

To further investigate the Am specific *N*^6^-methylation mechanism of METTL4, we solved the crystal structure of METTL4 complexed with 3-nucleotides (3-nt) Am-containing RNA oligo (5′-AAmG-3′) (Fig. 3a), a sequence taken from the Arabidopsis U2 snRNA around Am31, as a substrate in the presence of SAH (Table 2). In the complex structure, though only the central Am is visible in the electron-density map (Fig. 3d, e), we found the base of Am inserted into the catalytic pocket located in the turning corner of a L-shaped cavity with highly positive charges on the most conserved surface across the METTL4 homologs (Fig. 3a–c). There are four characteristic loops around the L-shaped cavity (Fig. 3d): Gate Loop1, Gate Loop2 and Interface Loop, named after the METTL3 structure^15^, and Entry Loop. The *N*^6^ atom of Am forms a tetrahedral like hydrogen network with the side chain of D233 and Y247, as well as the main chain O atom of P234 from Gate Loop1 (Fig. 3f, g). In addition, the *N*^7^ atom of Am forms a hydrogen bond with the side chain of S362 from Gate loop2, and the *N*^3^ atom of Am directly binds to the side chain of E325 (Fig. 3f, g). These interactions allow the *N*^6^ atom point to the sulfur atom of SAH with 4.7 Å, which is similar to the corresponding distance observed for the *N*^6^-adenine DNA methyltransferase M ▪*Taq*I (PDB code: 1G38)^35^. Notably, the dual conformations of Y247 were captured in the complexes, a free conformation obtained from the Am-absent complex and an active but restricted conformation obtained from the Am substrate-containing complex. Compared to the free conformation that flips out, the side chain of Y247 in active conformation turns ~131°towards the active site, resulting in the N6 atom of Am in the active site being attracted by the OH-group of Y247 (Supplementary Fig. 7d). Mutation of Y247 to alanine (Y247A) or phenylalanine (Y247F) and E325 to alanine (E325A) abolished the activity (Fig. 3h), highlighting the important role of these residues in placing Am base into the precise position for *N*^6^-methylation. The phosphate group of the Am is bound by the H321 from Interface Loop, and mutations of the positively charged residues, such as R278 in the Entry Loop and K322 in the Interface loop, impaired the enzymatic activity, suggesting that these positively charged residues in the four loops are important for substrate recognition.

### Specific recognition of Am-containing substrate by METTL4

**Fig. 4:**
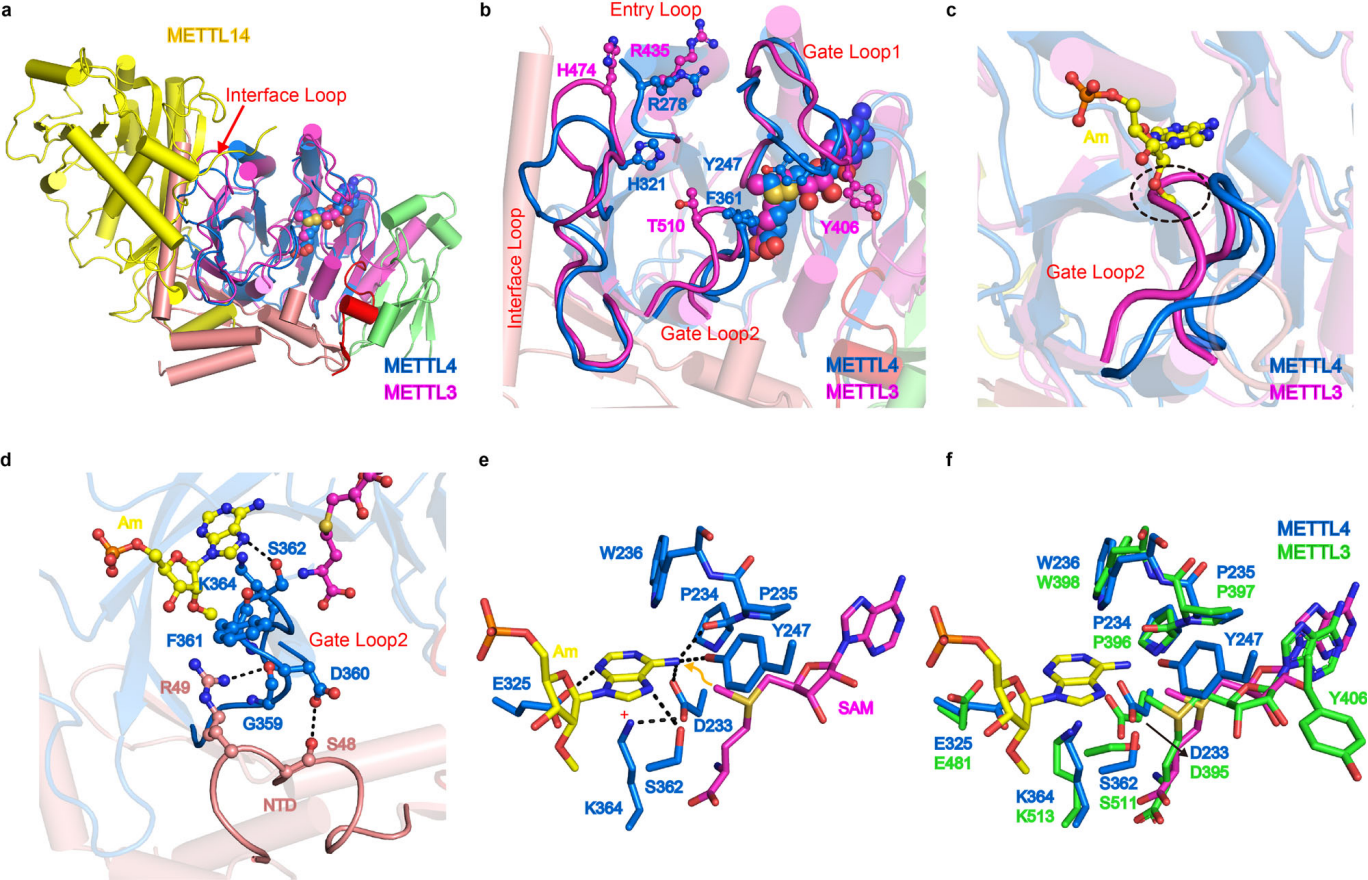
Structural comparison of METTL4 and METTL3/METTL14. **a** Superposition of the METTL4 with METTL3-METTL14 heterodimer. **b** Close-up view of the catalytic pocket formed by the four loops in the MTase domains of METTL4 and METTL3, and the key residues were shown as sticks. **c** Structural superposition of METTL4 and METTL3 shows the Gate Loop2 makes clashes with Am. **d** The NTD makes interaction with Gate Loop2 of MTase domain. **e** Modeled catalytic center of METTL4 by replacing SAH with SAM. **f** Structural superposition of the catalytic center of METTL4 and METTL3.

When we superimposed METTL4 with the METTL3-METTL14 complex, there are extensive clashes of the NTD domain with METTL14 (Fig. 4c), and the 2′-O-methyl (2′-OMe) of Am is clearly clashing with the Gate Loop2 even though most of residues involved in the Am interaction are conserved in METTL3 and METTL4 (Fig. 4d, e and Supplementary Fig. 8). Sequence alignment suggested that an aromatic residue F361 in Gate Loop2, which is relatively conserved in METTL4 proteins (Phe or His), provides a more hydrophobic environment for the methyl group. However, the residue Phe or His in Gate Loop2 of METLL4 is threonine (T) in METTL3, and mutation of F361A significantly reduced the activity (Fig. 3f), suggesting that Gate Loop2 may play a key role in distinguishing 2′-OMe and 2′-OH by METTL4. In addition, unlike the Interface Loop of METTL3, which is bound by METTL14 thus providing an RNA-binding scaffold^15^, the Interface Loop of METTL4 is stabilized by α4-helix in NTD (Figs. 2d and 3b), suggesting that NTD of METTL4 plays a similar role of METTL14 for METTL3. We note that the two highly conserved residues in NTD, S48, and R49, interact with D360 and G359 in Gate loop2, respectively (Fig. 4f), which stabilizes the conformation of Gate loop2 for the Am recognition and especially for the 2′-O-methyl group interaction (Supplementary Fig. 9). Consistently, mutations of R49 to alanine (A), asparagine (N) or negatively charged glutamic acid (E), dramatically reduced activity (Fig. 3f). These results suggest a critical role for NTD in the regulation of substrate recognition by METTL4.

### Conserved residues involved in binding *N*^*6*^ of adenosine

To further reveal the catalytic mechanism, we modeled the catalytic center by replacing SAH with SAM in the structure of the METTL4-Am-SAH complex (Fig. 4e), and noticed that K364, an absolutely conserved residue in Gate loop2 across MT-A70 family proteins (Supplementary Fig. 8), perpendicularly interacts with the Am base. It is plausible that the side chain of K364 forms cation-π interaction with the Am base in the catalytic center, when the amino group of K364 side chain forms a salt bridge ionic interaction with D233, which also forms a hydrogen bond with the *N*^6^ atom of the Am base (Fig. 4f). Interestingly, D233 interacts with SAM in the structure of the METTL4-SAM complex. Therefore, with the assistance of D233, K364 may function as a general base to promote the deprotonation of the *N*^6^ amino group of Am and catalyze the methyl group transfer from SAM to the *N*^6^ atom of adenosine. Consistently, mutation of K364 to alanine (K364A) or aspartic acid (K364D) abolished the catalytic activity (Fig. 3f). Superposition of the conserved residues in the modeled active site of METTL4 with corresponding residues in METTL3 clearly showed that most key residues are in the same conformation (Fig. 4f), suggesting that MT-A70 family proteins may share the novel common catalytic mechanism of *N*^6^ methylation of adenosine. The only exception is the residue Y247 in METTL4 that forms hydrogen bond with *N*^6^ of Am substrate, but the corresponding residue Y406 in METTL3 flips away without binding substrate, indicating that Y247 or Y406 may play a key role in recognition of *N*^6^ of substrate adenosine base.

### A proposed catalytic mechanism of Am by METTL4

**Fig. 5:**
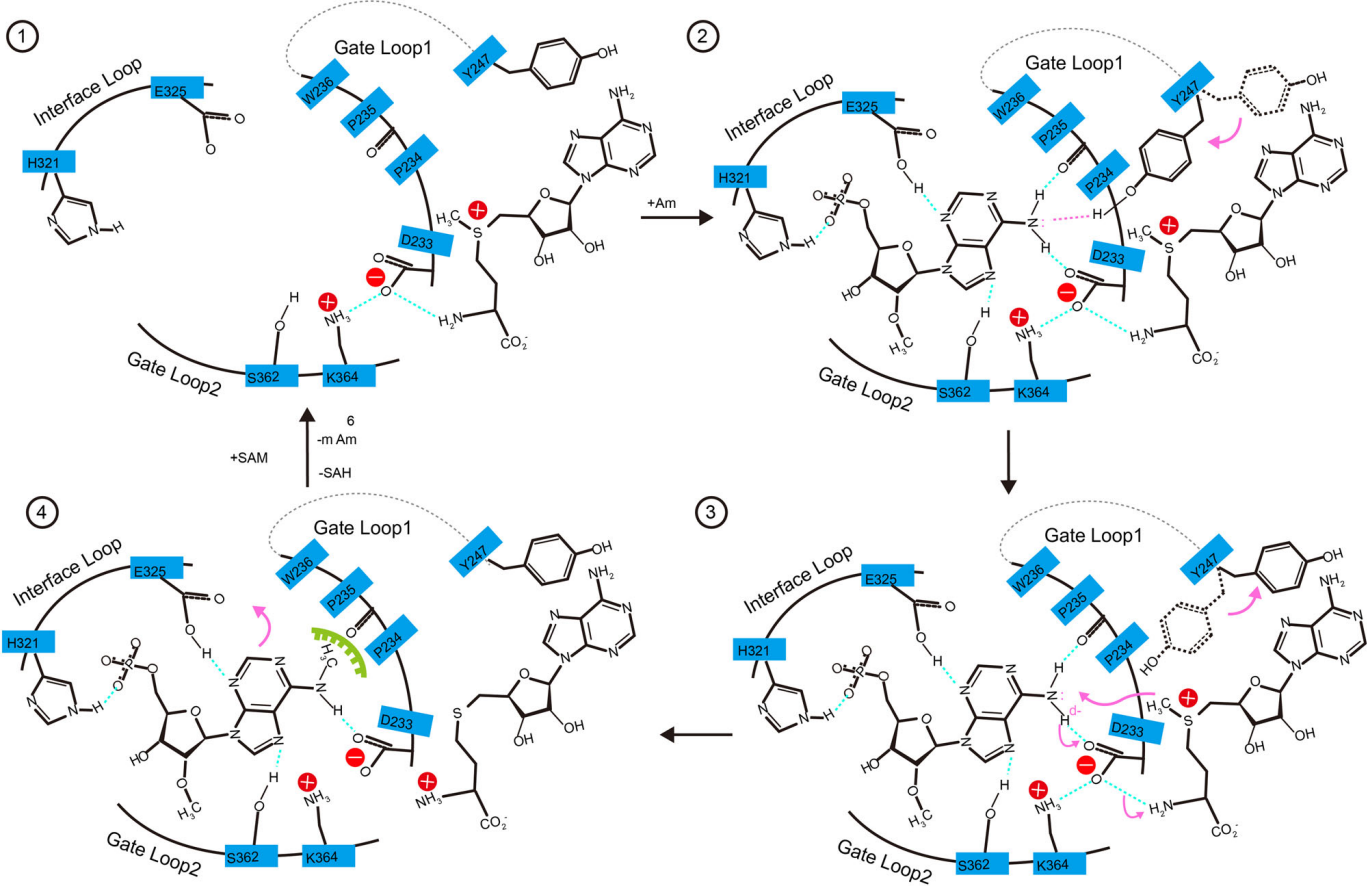
Proposed catalytic mechanism of METTL4. Diagrammatic presentation of the catalytic mechanism of METTL4. In the initial step (1) the backbone phosphate of Am may be bound by H321 in interface loop; then, Y247 in the Gate Loop1 interacts with *N*^6^ of Am (2), followed by hydrogen binding from D233 and P234 and methyl group transferred from SAM to *N*^6^ of Am (3). Finally, *N*^6^-methylated Am is released from the pocket due to the steric clash (4).

Based on structural and modeling studies, we propose a model for the catalytic progresses in the catalytic pocket (Fig. 5). First, the backbone of the target base is recognized by the Interface Loop, thus providing the opportunity to fit the base into the catalytic pocket. Second, the target base is stabilized via the interactions mediated by the 2′-O-methyl group with the Gate Loop2, and the hydrogen bond between *N*^6^ of adenosine base and Y247 guiding the *N*^6^ atom to an optimal catalytic position. Third, in the catalytic position, the *N*^6^ atom forms hydrogen bonds with the side chain D233 and the main chain P234 resulting in the deprotonation of *N*^6^ amine group, and the deprotonated *N*^6^ amine group attacks the activated methyl group of SAM. Fourth, the methyl group at the *N*^6^ atom makes extensive clashes with the D233 and P234, which promotes the *N*^6^-methylated adenosine base to flip out from the catalytic pocket.

## Discussion

This study demonstrated that the Arabidopsis METTL4, similar to human METTL4^20^, is responsible for m^6^Am modification of U2 snRNA in vivo and in vitro, and plays a similar role in the regulation of RNA alternative splicing. The crystal structure of the METTL4-Am-SAH complex in this study provided critical insights into the molecular mechanism for *N*^6^-adenosine methylation of an Am-containing RNA substrate. Based on the sequence and structural comparisons (Fig. 4c and Supplementary Fig. 8), the catalytic mechanism proposed here (Fig. 5) is probably shared by another MT-A70 family methyltransferase METTL3, which is consist with the phylogenetic analysis that METTL3 and METTL4 belong to the same MT-A70 clade^22^. The topologies of the MTase domains of METTL4 and METTL3 are similar but different from other RNA m^6^A or m^6^Am methyltransferases, such as METTL5^18^, ZCCHC4^36^, METTL16^37^, and CAPAM^38^. Unlike the aromatic reside F or Y in the conserved [D/N]PP[F/Y] motif, which functions to stabilize the target base in the catalytic pocket, the aromatic residue W236 in the DPPW motif in METTL4 is buried in a hydrophobic pocket adjacent to the Am binding site (Supplementary Fig. 9), implying that the catalytic mechanism of *N*^6^-methylation mediated by METTL4 is different from the previously proposed *N*^6^-methyl-ammonium cation mechanism for the *N*^6^-adenine DNA methyltransferase M▪*Taq*I^35^. Owing to the highly conserved residues in the active site of METTL4 and METTL3 (Fig. 4f), the adenosine base of the substrate is recognized by two MT-A70 family enzymes in a similar way. Therefore, the crystal structure and modeling study of METTL4 in complex with the substrate may help to design more specific inhibitors of METTL3 in developing drugs as a therapeutic strategy against m^6^A-associated human diseases^39^.

The Am-containing RNA substrate is bound by an L-shaped, positively charged cavity formed by four loops in METTL4, which are also conserved in METTL3. However, the Interface Loop in METTL4 is bound by a long helix from NTD, which replaces METTL14 that stabilizes METTL3 and coordinates with METTL3 to form a substrate binding cavity^15^. Therefore, METTL4 functions as a monomer (Supplementary Fig. 6c), but METTL3 forms a functional heterodimer with METTL14^15^. Another difference is that METTL4 prefers Am while METTL3 prefers A instead of Am as the substrate, although Drosophila METTL4 appears to act on A in U2 snRNA in vivo^22^, maybe due to the variation in sequences within Gate Loop2 (Supplementary Fig. 4). Lastly, the sequences of NTD are less conserved in METTL4 proteins across diverse species (Supplementary Fig. 4), which may account for their different preferences for 2′-OH or 2′-OMe of the adenosine substrates. Further study is needed for the Am preference by METTL4 from the evolutionary perspective.

## Methods

### METTL4 construction, expression, and purification

The open reading frame (ORF) of *METTL4* (AT1G19340) was amplified from an *Arabidopsis thaliana* cDNA library and the ORF segment was cloned into the pET28vector containing a SUMO tag between *Nde* I and *BamH* I. The protein was expressed in *E.coli* BL21(DE3) and the cells were induced at OD_600_ ~0.6 by isopropyl β-d-1-thiogalacto-pyranoside (IPTG) with a final concentration of 0.2 mM for 20 h at 14 °C. Cells were harvested by centrifugation at 6000 rpm for 15 min and homogenized in ice-cold buffer1 containing 20 mM Tris-HCl, pH 8.0, 500 mM NaCl and 25 mM imidazole. The cells were crushed by high pressure machine and followed by ultracentrifugation at 18,000 rpm for 1 h at 4 °C. The supernatant was loaded onto a Ni-NTA column (GE healthcare) and the sample was eluted by gradient using buffer2 (20 mM Tris-HCl, pH 8.0, 500 mM NaCl and 500 mM imidazole). The tag was cleaved by ulp1 protease and dialyzed against buffer containing 20 mM Tris-HCl, pH 8.0 and 500 mM NaCl. Then, sample was loaded onto a Ni-NTA column again and collected the flow through, and the sample was future purified by Superdex 200 16/600 column (GE healthcare) equilibrated by buffer containing 20 mM Tris-HCl, pH 8.0, 100 mM NaCl and 5 mM DTT. The peak fraction was concentrated to about 30 mg/ml using Amicon 30-kDa cutoff (Millipore). The mutations were generated by overlap PCR method and the purification of mutated proteins was the same as the wild-type.

### Crystallization

The initial crystallization was performed by Gryphon crystallization robot system using sitting-drop vapor diffusion by mixing 0.2 μl protein sample (6 mg/ml) with 0.2 μl mother solution at 18 °C and crystallization kits from the Hampton research company. All optimization procedures were used the hanging-drop vapor diffusion method at 18 °C. For METLL4 complex, the protein was incubated with 1 mM SAM (S-adenosylmethionine purchased from Sigma, CAS number: 86867-01-8), or SFG (sinefungin, purchased from SANTA CRUZ BIOTECHNOLOGY, CAS number: 58944-73-3) or SAH (S-adenosyl-L-homocysteine purchased from Sigma, CAS number: 53186-57-5) in final concentration. The crystals of Se-METLL4 grew in the condition including 0.1 M tacsimate pH5.5 and 8% PEG3350, and the crystals of METTL4-SAM complex grew in the condition including 0.1 M sodium malonate pH5.5 and 6% PEG3350. The crystal of METTL4 bound to SFG were grown in the condition of 1 M Lithium chloride 0.1 M HEPES pH 7.0 10% PEG6000, and the crystal of METTL4 bound to SAH was obtained from the condition of 1600 mM Magnesium sulfate 100 mM MES/ Sodium hydroxide pH6.5. For the complex of METTL4-Am-SAH, the crystal of METTL4-SAH grew in the condition consist of 0.49 M NaH_2_PO_4_ and 0.91 M K_2_HPO_4,_ and the crystals were soaked with 10 mM RNA oligo (5′-AAmG-3′) at final concentration, and the RNA oligo (5′-AAmG-3′) was synthesized and purified in Cao’s lab at Shanghai Institute of Organic Chemistry, Chinese Academy of Sciences. For the METTL4-DNA-SAH complex, the synthetic DNA sequence 5′-GCCGCGTGATCACGCGGC-3′ was annealed and incubated with METTL4 (6 mg/ml) with the molar ratio 1.1:1.0. The crystals were grown in the condition including 0.1 M sodium malonate pH5.0 and 12% PEG3350.

### Data collection and structure determination

All crystals were transferred to a cryoprotectant solution containing mother liquor and 20% glycerol. All data were collected at the shanghai synchrotron radiation facility (SSRF) on beamlines BL17U, BL18U, or BL19U using a CCD detector cooled under liquid nitrogen. The data of selenomethionine-labeled METTL4 (Se-METTL4) crystals were collected by multiwavelength anomalous dispersion (MAD), and the data of METTL4-SAM, METTL4-SAH, METTL4-SFG and METTL4-Am-SAH complex were collected by single wavelength anomalous dispersion (SAD) and processed with HKL2000 or HKL3000 program. The structure of METTL4-SAM, METTL4-SAH, METTL4-SFG, and METTL4-Am-SAH, METTL4-DNA-SAH were solved by molecular replacement of ccp4i program using the Se-METTL4 as a search model. All crystal structures were built using Coot^40^ and refined by ccp4^41^ and Phenix^42^ program. The conservation of the surface of METTL4 was analyzed by ConSurf^43^, and the diagrams of interactions involving in Am and SAM were analyzed by LigPlot^44^. All structure pictures representing in paper were prepared with PyMOL.

### ITC

ITC experiments were performed at 25 °C using a MicroCal iTC200 microcalorimeter. Before the reactions, proteins and SAM were dialyzed against buffer containing 100 mM NaCl and 10 mM HEPES (pH 7.5). In all, 3 mM SAM with an initial injection volume of 0.5 μL (omitted from the analysis) followed by 19 injections (each 2 μL) were titrated into 150 μM proteins. Data were analyzed using Origin 7 software, and the heat of dilution was subtracted from the raw values. Dissociation constant (*K*_d_) values were calculated by fitting the isotherm.

### Plant materials

Arabidopsis *mettl4-1* mutant was obtained from the Saskatoon collection (http://aafc-aac.usask.ca/FST/), and all *Arabidopsis thaliana* strains used in this study were in the Columbia (Col) background. Plants grown under long-day (LD) condition (22 °C and 16-h-light/8-h-dark) were used for flowering phenotype analysis. For RNA extraction, plants were cultured for 14-days on agar-solidified Murashige and Skoog medium M0255 (Duschefa) supplemented with 0.9% sucrose at 21 °C under a 16-h-light/8-h-dark photoperiod in a Percival AR41L5 growth chamber in 60 to 90 μmol m^−2^ s^−1^ white light. The *mettl4-1* mutant was rescued respectively by introducing *P*_*METTL4*_*::HA-METTL4-Flag* and *P*_*METTL4*_*::HA-METTL4(D233A/W236A)-Flag* constructs, in which HA- and Flag-tagged wild-type METTL4 and mutated METTL4 (D233A/W236A) were expressed under *METTL4* native promoter.

### U2 snRNA purification

Total RNA was extracted from 12-day seeding Arabidopsis by a standard method using Trizol. A synthetic oligo complementary to the Arabidopsis U2 snRNA (5′-biotin-GATACTACACTTGATCTTAGCCAAAAGGCCGAGAA, 500 ng) were incubated with total RNA (10 μg), in 50 μL hybridization buffer (150 mM NaCl, 50 mM Tris-HCl, pH 7.9). The hybridization mixture was incubated at 90 °C for 7 min, and slowly cooled to room temperature (25 °C) to allow hybridization to occur. The annealed products were mixed with Dynabeads M-280 Streptavidin (500 μg, Invitrogen, 11205D) at room temperature for 30 min. The annealed probes were mixed with Dynabeads M-280 Streptavidin (500 μg, Invitrogen, 11205D) at room temperature for 30 min. Place the mixture on magnet until the liquid become clear, then remove and collect the flow through (the flow through was called as U2 RNA removal in Fig. 1f). After that, wash the beads with 50 μl hybridization buffer for twice. And the sample were eluted in nuclease-free H_2_O by heating at 70 °C for 5 min. The elution and flow through were used for following digestion and UHPLC-MS/MS analysis.

### RNA digestion

For the in vitro enzyme assay: The probes were dissolved into nuclease-free H_2_O and annealed by heating briefly to 95 °C for 5 min, followed by gradually cooling to 4 °C in PCR machine.

Each probe (90 pmol) was incubated with 4 μg METTL4 protein in 30 μl reaction buffer (0.8 mM d3-SAM, 10% glycerol, 10 mM Tris-HCl pH 7.0) at 28 °C for 1 h. After reaction, all sample were desalted and purified by ice ethanol precipitation. The recovered probes or Arabidopsis total RNA were digested into single nucleosides by 0.5 U Nuclease P1 (NP1, Sigma, N8630) and 0.5 U alkaline phosphatase (CIP, New England Biolabs, M0290) in 50 µL 10 mM Tris-HCl (pH 7.6) reaction volume at 37 °C for 4 h. The digestive enzyme in the samples were removed by ultrafiltration with 3 kD ultrafiltration tubes (Pall Corporation, Port Washington), and then subjected to UHPLC-MS/MS analysis.

### UHPLC-MS/MS

The UHPLC-MS/MS analysis was performed on an ultra-high performance LC system (Agilent 1290 II) coupled with G6495 mass spectrometer. 5 μl sample was injected in to the UHPLC system and the nucleosides were separation by C18 column (Zorbax Eclipse Plus C18, 2.0 mm×100 mm i.d, 1.8 μm particle size, Agilent Technologies, Palo Alto, CA). All the targets were separated in HPLC condition with mobile phase consisted of two solvents: 0.1% formic acid in water (A), and 100% in methanol (B). Set up following separation condition with the gradient elution: 0-2 min, 5% B; 2–4 min, 30% B; 4-6 min, 30% B, 6–9 min, 5% B, and the flow rate was 0.25 mL/min. For the mass spectrometry parameters, the fragmentation voltage was set at 90 V; using nitrogen for nebulization and desolvation, and the flow rate and temperature of gas were set at 9.0 L/min and 300 °C, respectively; collision gas used high purity of nitrogen with 99.999%. The multiple reaction monitoring (MRM) mode was chosen to quantify all the targets: m/z 292 → 150 for m^6^Am, m/z 282 → 150 for m^6^A and m^1^A; m/z 282 → 136 for Am, m/z 266 → 150 for ^6^mA. The collision voltage of all targets was set to 5 eV. All the sample were repeated at three times.

### RNA sequencing

Total RNA was extracted from 14-days seedlings using an RNAprep pure Plant Kit (Tiangen Biotech) under the guidance of manufacturer’s instructions. A strand-specific RNA-seq library was constructed according to the KAPA stranded mRNA-seq Kit instructions (Illumina® Platforms). There were two replicates for WT and *mettl4-1*.

Raw RNA-seq paired-end reads were filtered to remove the adapters and reads length less than 20 bp and reads with low sequencing base quality score using Cutadapt (version 3.5)^45^. The filter reads were mapped to the Arabidopsis thaliana genome (TAIR10) by Hisat2 (version 2.2.1)^46^. High mapping quality reads were obtained using SAMtools (version 1.13)^47^. The track files which fragments were normalized as fragments per bin (bin size = 10 bp) per million mapped fragments were obtained using bamCoverage tool in deepTools (version 3.5.1)^48^ and viewed with IGV (version 2.11.2)^49^. FeatureCounts (version 2.0.1) was used to calculate the read number for each gene^50^. DESeq2 (version 1.32.0) was used to identify differentially expressed genes (DEGs)^51^. Compared to the mutant and wild-type, genes that showed |log2foldchange|≥ log2(1.5) and *p* value < 0.05 were considered to be DEGs. Gene ontology (GO) enrichment analysis was carried out with DAVID^52^ and ClusterProfiler^53^. Different alternatively splicing genes between the mutant and the wild-type (threshold of |Δψ| > 0.1, *p* value < 0.05) were identified by rMATS (version 4.1.1)^54^.

### Reporting summary

Further information on research design is available in the Nature Research Reporting Summary linked to this article.

## Supplementary information

Supplementary Information

Reporting Summary

## Data Availability

The atomic coordinates of the X-ray crystal structures have been deposited to the Protein Data Bank with following accession codes 7CVA for apo-METTL4, 7CV7 for METTL4-SAM, 7CV9 for METTL4-SAH, 7CV8 for METTL4-SFG, and 7CV6 for METTL4-Am-SAH. The RNA-sequencing data reported in this study have been deposited in NCBI’s Gene Expression Omnibus with the accession number GEO Series accession number GSE190241. Source data are provided with this paper.

## Source data

Source Data
