# Supplementary material for: Structural insights into molecular mechanism for N6-adenosine methylation by MT-A70 family methyltransferase METTL4
Source: Nat Commun. 2022 Sep 26;13:5636. doi: 10.1038/s41467-022-33277-x (PMC9512776; doi:10.1038/s41467-022-33277-x)
Supplement: Supplementary file 1 — Supplementary Information [file 41467_2022_33277_MOESM1_ESM.pdf]

Supplementary Information

Structural insights into molecular mechanism for N^6-adenosine methylation by MT-A70 family methyltransferase METTL4

Qiang Luo[1,3,†], Jiezhen Mo[2,†], Hao Chen[4,†], Zetao Hu[1], Baihui Wang[3], Jiabing Wu[3], Ziyu Liang[2], Wenhao Xie[3], Kangxi Du[3], Maolin Peng[3], Yingping Li[3], Tianyang Li[3], Yangyi Zhang[4], Xiaoyan Shi[4], Wen-Hui Shen[3,5], Yang Shi[6*], Aiwu Dong[3*], Hailin Wang[2,7*], Jinbiao Ma[1*]

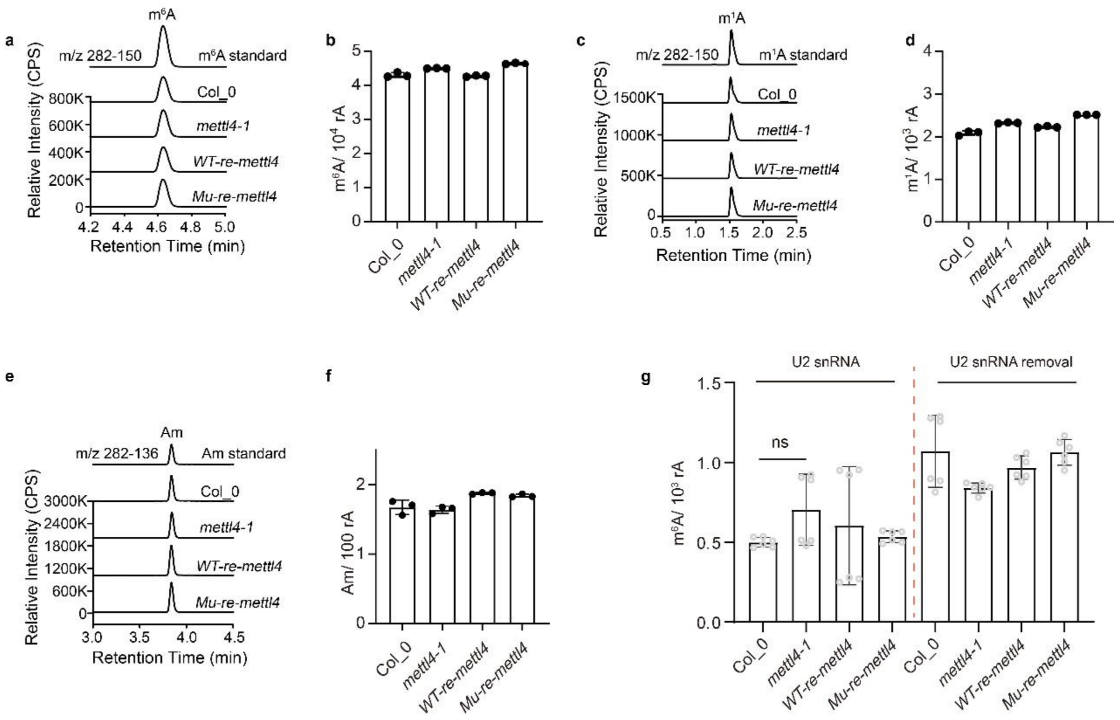

Supplementary Fig.1| Detection the RNA modification changes in wild-type and mutants. **a.** UHPLC-MRM-MS/MS chromatograms of m^{6}A modification in total RNA and quantification (**b**). **c.** UHPLC-MRM-MS/MS chromatograms of m1A modification in total RNA and quantification (**d**). **e.** UHPLC-MRM-MS/MS chromatograms of Am modification in total RNA and quantification (**f**). **g**. The quantification of m^{6}Am abundance in U2 snRNA and residual RNAs.

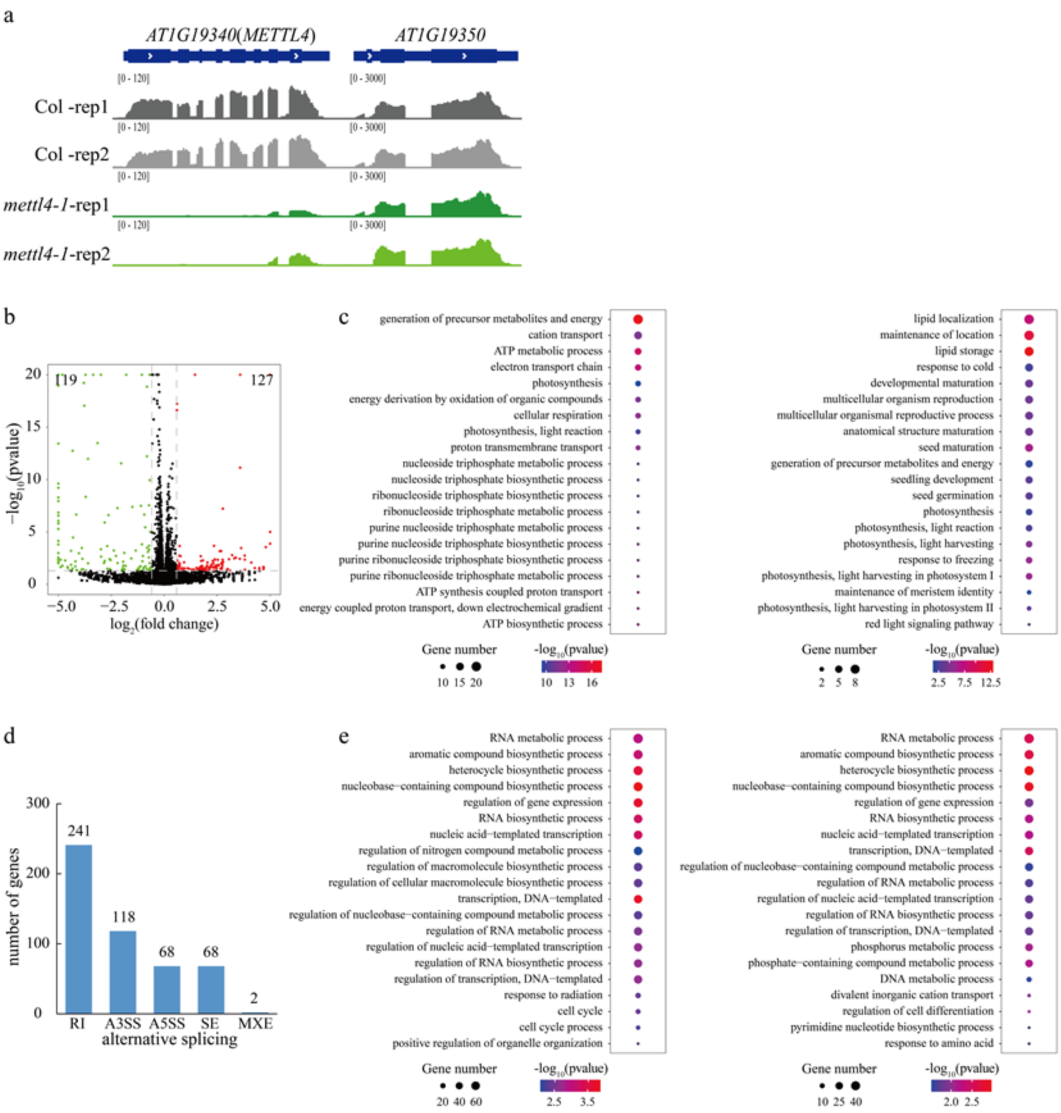

Supplementary Fig.2| Transcriptome analysis of the *METTL4* knockout mutant. (a): IGV screen shots presenting the normalized read densities of *METTL4* and its adjacent gene based on an RNA-sequencing analysis of two biological replicates of the wild-type Col-0 (WT) and the *mettl4-1* mutant. (b): Volcano plot presenting gene transcription changes following the knockdown of *METTL4*. Green and red points represent the genes exhibiting down- and up-regulated expression (> 1.5-fold change, $p < 0.05$), respectively, whereas the black points represent the genes with unchanged expression, in the *mettl4-1* mutant compared with WT. (c): Functional enrichment

analysis of genes with significantly up- (left) or down-regulated (right) expression levels. The size of each point represents the number of genes, and p values are indicated with various colors. (d): The histogram shows the numbers of genes with alternatively splicing change in the *mettl4-1* mutant compared with WT (threshold of $|\Delta\psi| > 0.1$, $p < 0.05$). RI: retained intron; A3SS: alternative 3' splice site; A5SS: alternative 5' splice site; SE: skipped exon; MXE: mutually exclusive exons. (e): Functional enrichment analysis of genes with alternatively splicing (left) and RI changes (right). The size of each point represents the number of genes, and p values are indicated with various colors.

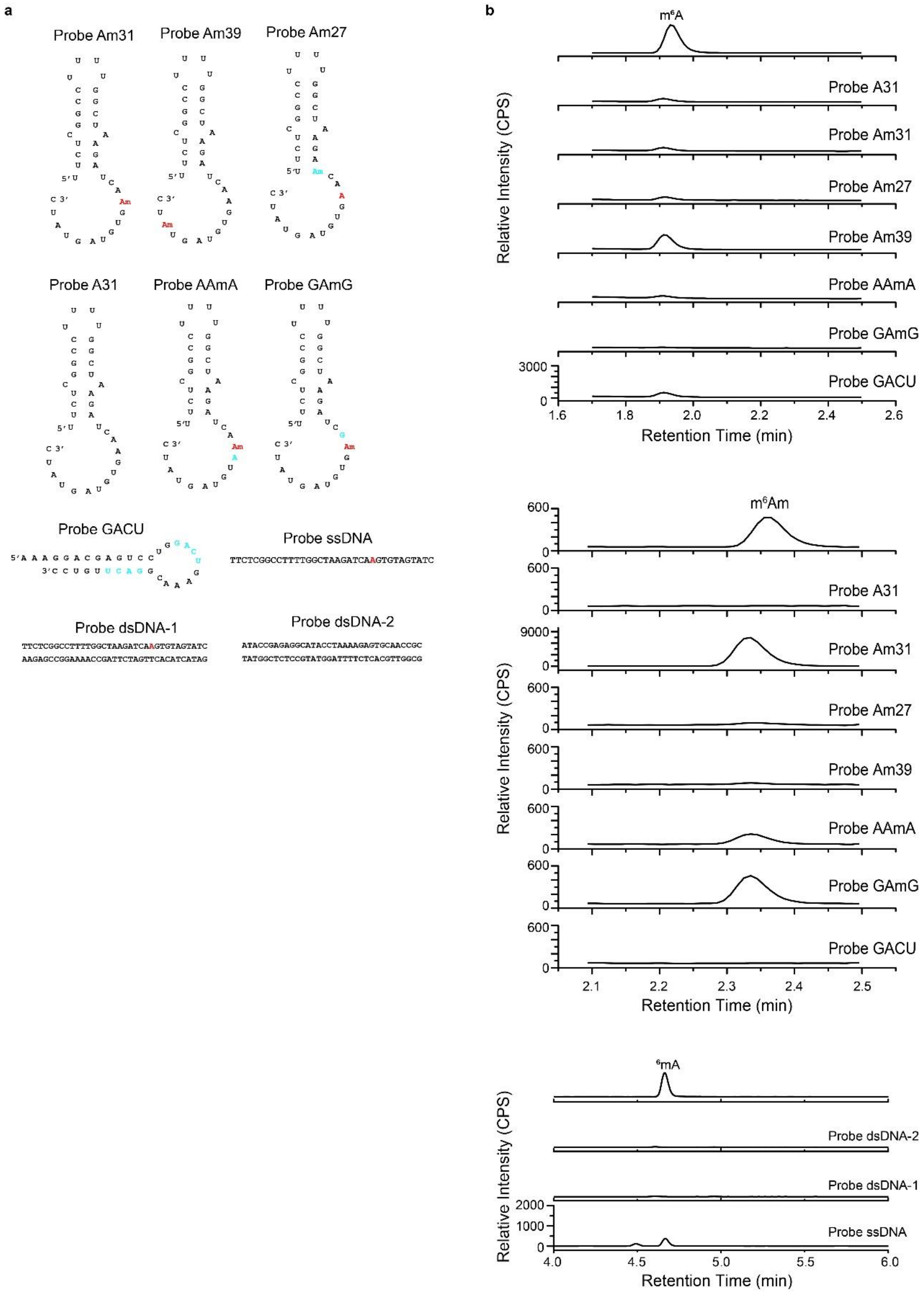

Supplementary Fig.3| Enzyme activity of METTL4 for different substrates. **a.** The fragments of Arabidopsis U2 snRNA for enzyme activity. **b.** Chromatograms of

METTL4 m^{6}Am RNA and ^{6}mA DNA methylation activity *in vitro* assay obtained from UHPLC-MS/MS using the fragments of (**a**).

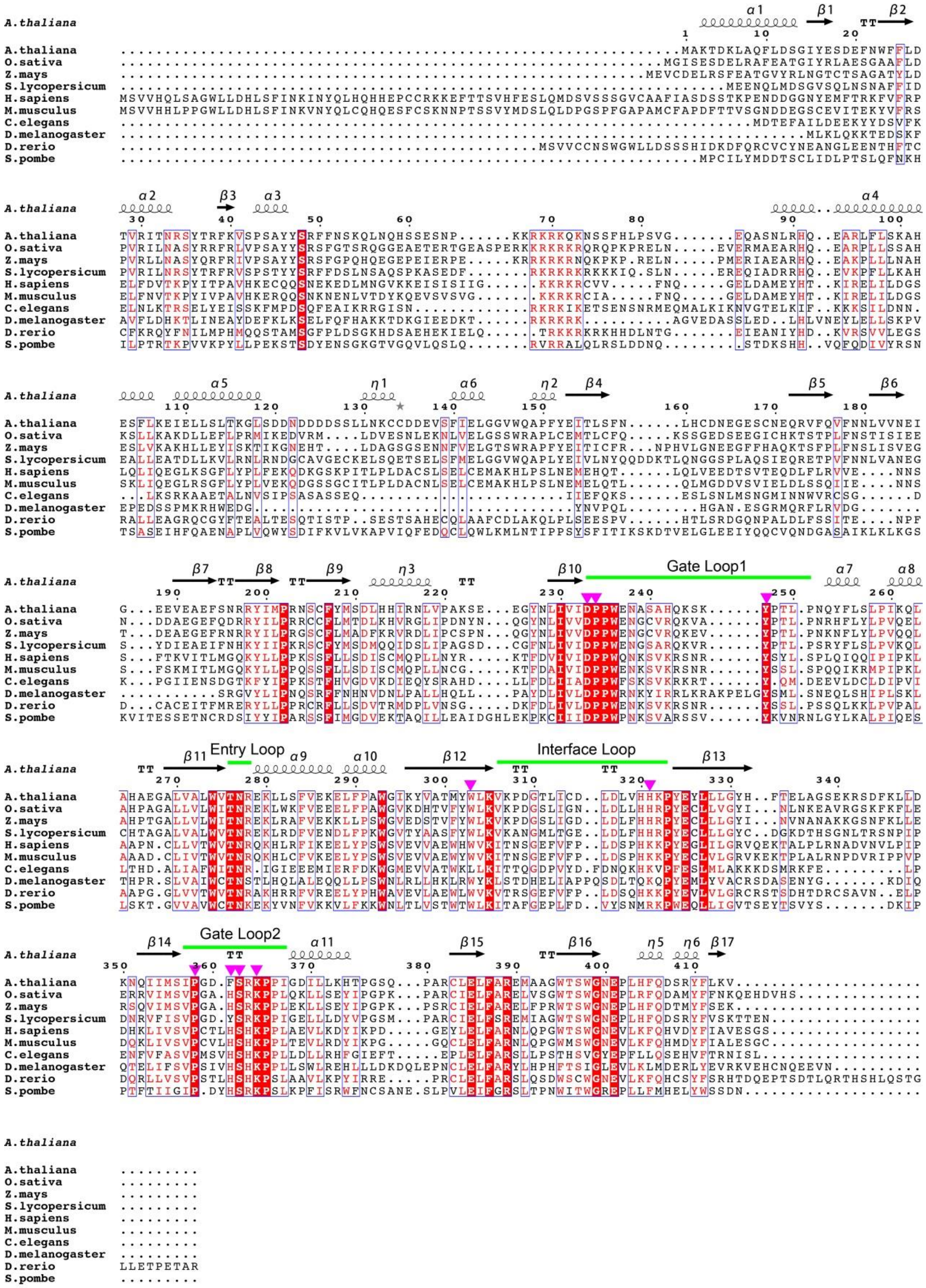

Supplementary Fig.4| Sequence alignment of METTL4 from different species. Residues involved in Am binding are indicated by magenta arrow.

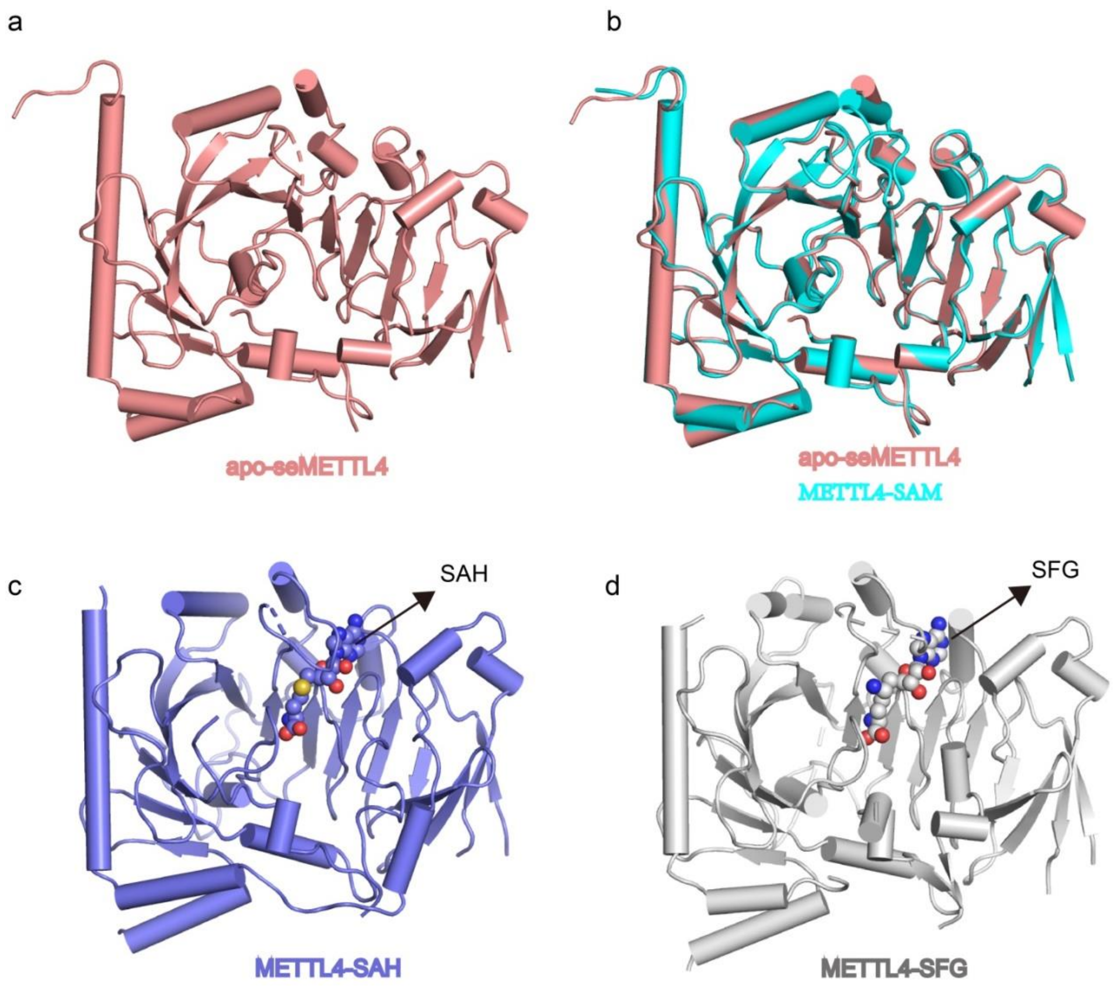

Supplementary Fig.5| Crystal structures of METTL4 bound to different cofactors. **a.** The apo-structure of SeMETTL4. **b.** Structure superposition of METTL4 bound to SAM with apo-structure of Se-METTL4. **c-d.** Crystal structure of METTL4 bound to SAH (**c**) and SFG (**d**), respectively.

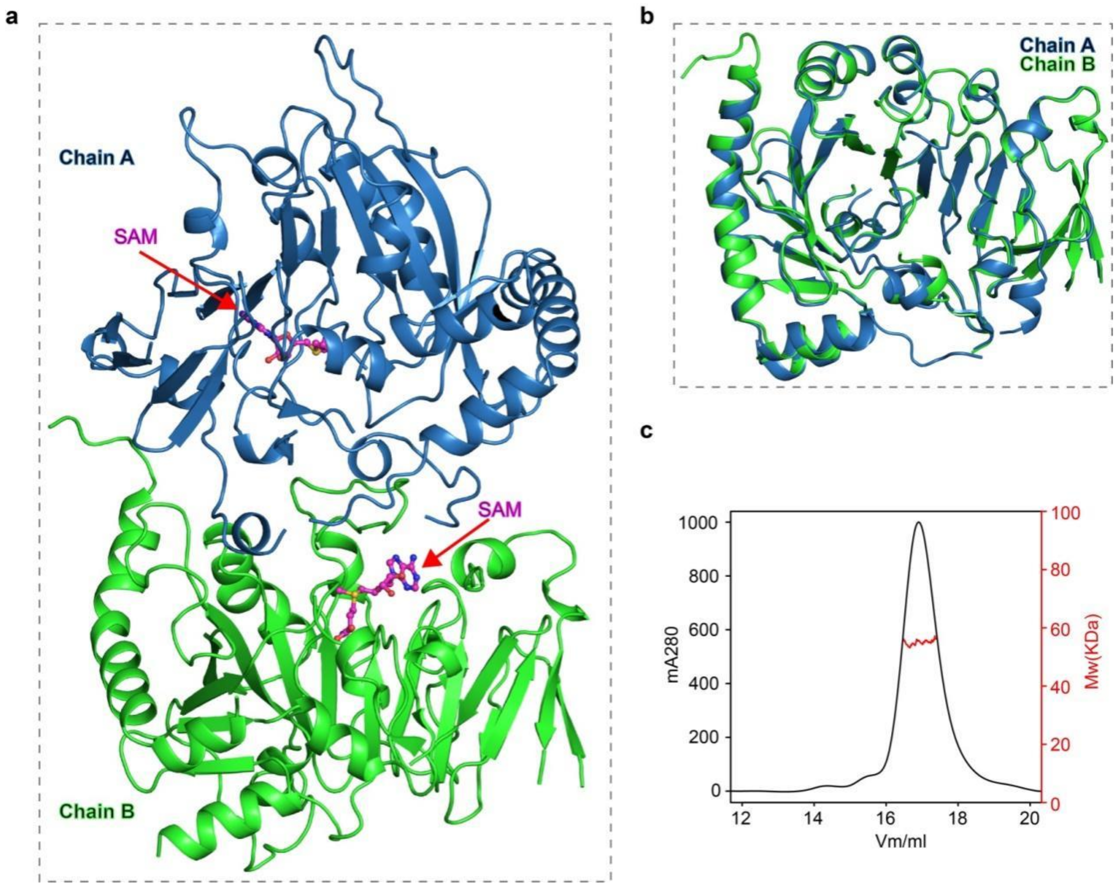

Supplementary Fig.6| Crystal structures of METTL4-SAM complex. **a.** The asymmetric unit of METTL4-SAM complex, and the chain A and chain B are shown as blue and green, respectively. **b.** Structure superposition of the chain A and chain B in (**a**). **c.** The molecular weight of METTL4 in solvent was measured by the elution profile of analytical SEC-MALS. The measured molecular weight of METTL4 is 54.7 KDa which is similar to the theoretical weight of 47.9 KDa.

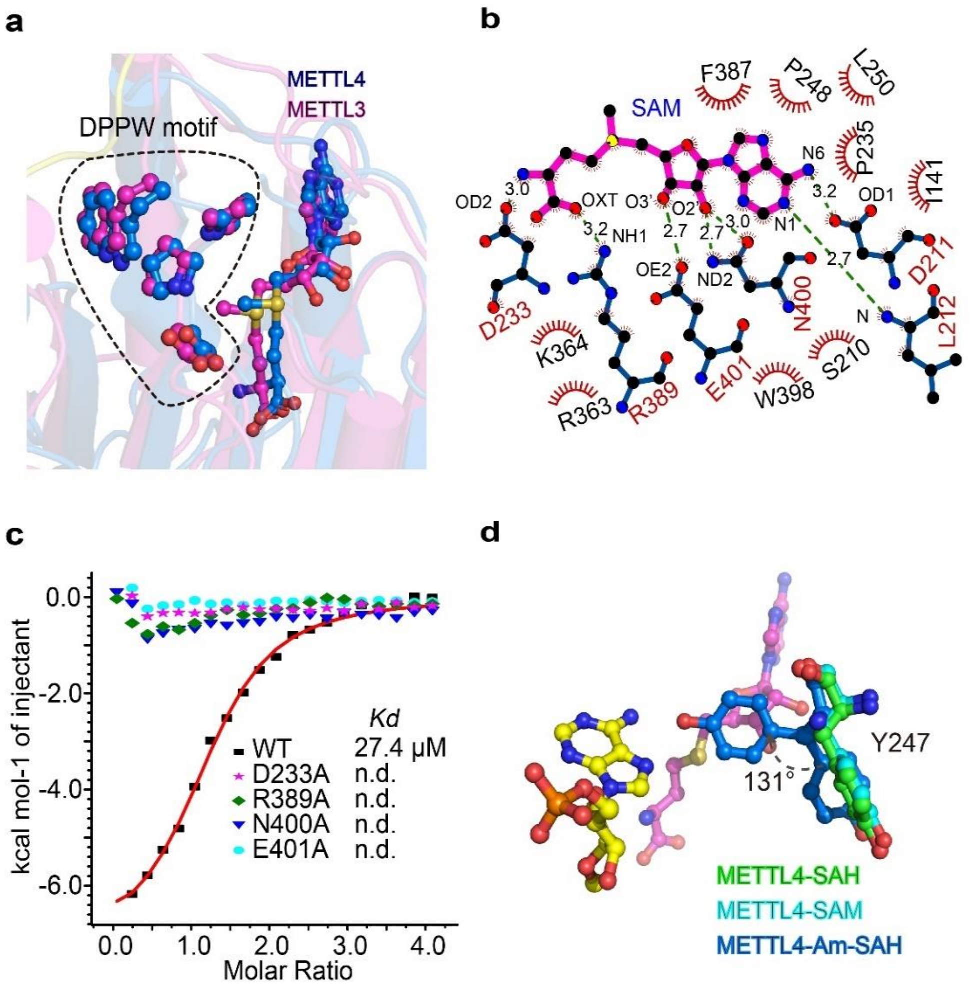

Supplementary Fig.7| Interaction between METTL4 and SAM. **a.** Structural superposition of METTL4-SAM and METTL3-SAM shows the SAM binding pocket is near to the conserved DPPW motif. **b.** The interactions between SAM and MTase domain was analyzed by LigPlot. **c.** Mutational assay of METTL4 binding to SAM measured by ITC experiment. **d.** The conformation changes of Y247 in METTL4 bound to Am compared to the structure of METTL4-SAH and METTL4-SAM.

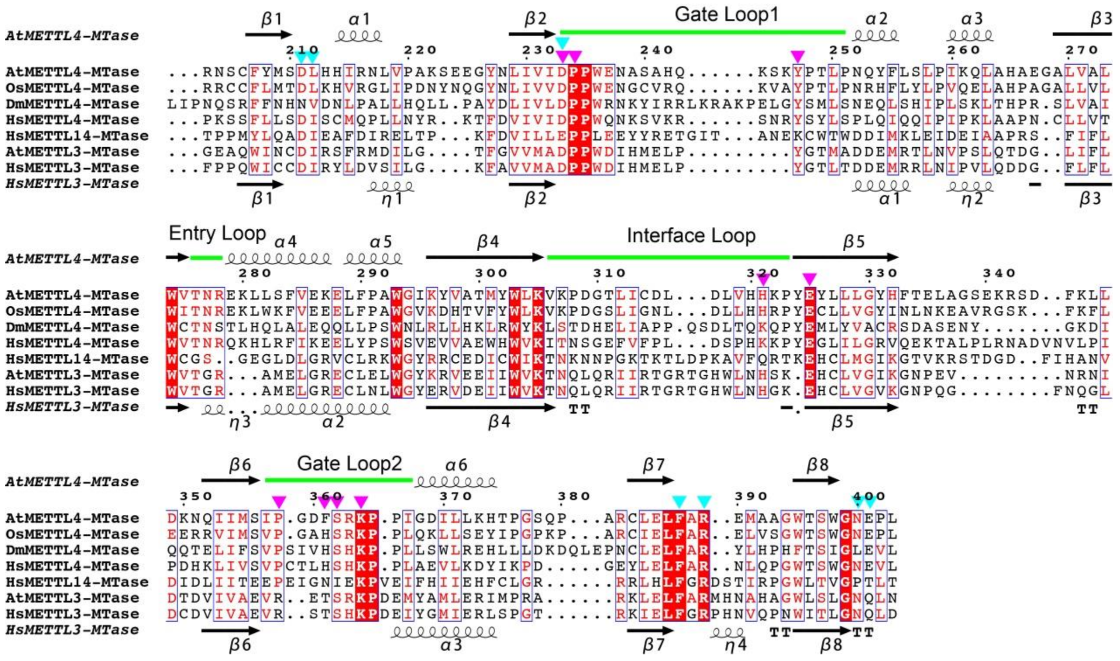

Supplementary Fig.8| Sequence alignment of the MTase domain from METTL4, METTL3 and METTL14 from different species, and the residues involved in Am interaction observed in METTL4 are indicated by magenta triangle. Residues involved in SAM binding are indicated by cyan triangle.

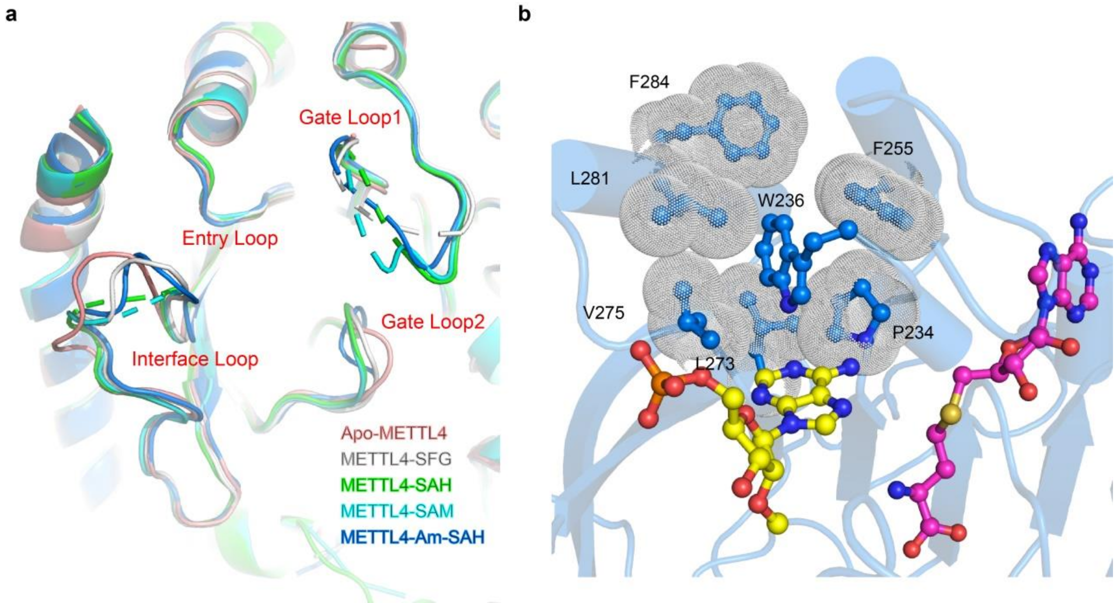

Supplementary Fig.9| Superposition of apo-METTL4 and METTL4 bound to SAM and different analogues. **a.** Superposition of the four loops of apo-METTL4 and METTL4 bound to SAM and different analogues. **b.** The conserved aromatic residues W236 from the DPPW motif is buried by a hydrophobic pocket.

Supplementary Table 1 Primers used in this study

Name	Sequence (5’ -3’)
**Primers for transgeneic plant**	
Promoter-F	ggtacccagccaatgccaaccaccgag
Promoter-R	ggcggatccttaacggctccaacactctctag
HA-METTL4-F	cgggatccatg tacccatacgatgttccagattacgctatggcgaaaactgataagct
FLAG-METTL4-R	gcgtcgacctatttgtcgtcatcgtctttgtagtcaac
APPA-F	gcaccgcctgcagaaaatgcaagtgctcatcag
APPA-R	ctgcaggcggtgcaataactataagattgtagccttcttcag
**Primmers for RT-PCR**	
METTL4-F	atggcgaaaactgataagctagctc
METTL4-R	ctaaactttcaaaaagtatctcgagtcctg
ACTIN2-F	ccctcagcacattccagcagatgt
ACTIN2-R	tgtgaacgattcctggacctgcct
**Primers for protein expression**	
METTL4-BamHI -F	cgcggatccatggcgaaaactgataagctagctcaattccttgattc
METTL4-SalI -R	acgcgtcgacttactaaactttcaaaaagtatctcgagtcctgaaag
P358S362/DE-F	gaaccagataatcatgagcatcgatggtgattttgagaggaaacccccaattggagata
P358S362/DE-R	tatctccaattgggggtttcctctcaaaatcaccatcgatgctcatgattatctggttc
K364D-F	gcatccctggtgatttttcgagggacccccccaattggagatatacta
K364D-R	tagtatatctccaattgggggggtccctcgaaaaatcaccagggatgc
D233A-F	ggctacaatcttatagttattgctccgccttgggaaaatgcaagt
D233A-R	acttgcattttcccaaggcggagcaataactataagattgtagcc
N400A-F	gccggatggacctcttggggagccgaaccgcttcactttcaggac
N400A-R	gtcctgaaagtgaagcggttcggctccccaagaggtccatccggc
E401A-F	cggatggacctcttggggaaacgcaccgcttcactttcaggactcga
E401A-R	tcgagtcctgaaagtgaagcggtgcgtttccccaagaggtccatccg
Y247A-F	gctcatcagaaatcaaaggctcctactttaccgaaccaa
Y247A-R	ttggttcggtaaagtaggagcctttgatttctgatgagc
F361A-F	tcatgagcatccctggtgatgcttcgaggaaacccccaattgg
F361A-R	ccaattgggggtttcctcgaagcatcaccagggatgctcatga
R278A-F	ggctttgtgggtgacaaatgcagagaaattactaagttttg
R278A-R	caaaacttagtaatttctctgcatttgtcacccacaaagcc
H320A-R	ggtactcataaggtttatgagcgaccaggtccaggtcgcaaatc

H320A-R	ggtactcataaggtttatgagcgaccaggtccaggtcgcaaatc
H321A-F	gcgacctggacctggtccatgctaaaccttatgagtaccttcta
H321A-R	tagaaggtactcataaggtttagcatggaccaggtccaggtcgc
K322A-F	acctggacctggtccatcatgcaccttatgagtaccttctacta
K322A-R	tagtagaaggtactcataaggtgcatgatggaccaggtccaggt
R389A-F	cggtgccttgagctatttgcggcggaaatggctgccggatggacc
R389A-R	ggtccatccggcagccatttccgccgcaaatagctcaaggcaccg
R49K-F	gtttcaccttctgcttactactctaagttcttcaattcgaagcaactcaat
R49K-R	attgagttgcttcgaattgaagaacttagagtagtaagcagaaggtgaaac
R49N-F	gtttcaccttctgcttactactctaatttcttcaattcgaagcaactcaat
R49N-R	attgagttgcttcgaattgaagaaattagagtagtaagcagaaggtgaaac
R49E-F	gtttcaccttctgcttactactctgaattcttcaattcgaagcaactcaat
R49E-R	attgagttgcttcgaattgaagaattcagagtagtaagcagaaggtgaaac
R49A-F	gtttcaccttctgcttactactctgctttcttcaattcgaagcaactcaat
R49A-R	attgagttgcttcgaattgaagaaagcagagtagtaagcagaaggtgaaac

83
