# Supplementary material for: Structural insights into molecular mechanism for N6-adenosine methylation by MT-A70 family methyltransferase METTL4
Source: Nat Commun. 2022 Sep 26;13:5636. doi: 10.1038/s41467-022-33277-x (PMC9512776; doi:10.1038/s41467-022-33277-x)
Supplement: Supplementary file 2 — Reporting Summary [file 41467_2022_33277_MOESM2_ESM.pdf]

nature research

Corresponding author(s): Jinbiao Ma

Last updated by author(s): Jul 10, 2022

Reporting Summary

Nature Research wishes to improve the reproducibility of the work that we publish. This form provides structure for consistency and transparency in reporting. For further information on Nature Research policies, see our Editorial Policies and the Editorial Policy Checklist.

Statistics

For all statistical analyses, confirm that the following items are present in the figure legend, table legend, main text, or Methods section.

n/a	Confirmed	
☐	☒	The exact sample size (*n*) for each experimental group/condition, given as a discrete number and unit of measurement
☐	☒	A statement on whether measurements were taken from distinct samples or whether the same sample was measured repeatedly
☐	☒	The statistical test(s) used AND whether they are one- or two-sided<br>*Only common tests should be described solely by name; describe more complex techniques in the Methods section.*
☒	☐	A description of all covariates tested
☒	☐	A description of any assumptions or corrections, such as tests of normality and adjustment for multiple comparisons
☐	☒	A full description of the statistical parameters including central tendency (e.g. means) or other basic estimates (e.g. regression coefficient) AND variation (e.g. standard deviation) or associated estimates of uncertainty (e.g. confidence intervals)
☒	☐	For null hypothesis testing, the test statistic (e.g. *F*, *t*, *r*) with confidence intervals, effect sizes, degrees of freedom and *P* value noted<br>*Give P values as exact values whenever suitable.*
☒	☐	For Bayesian analysis, information on the choice of priors and Markov chain Monte Carlo settings
☒	☐	For hierarchical and complex designs, identification of the appropriate level for tests and full reporting of outcomes
☒	☐	Estimates of effect sizes (e.g. Cohen's *d*, Pearson's *r*), indicating how they were calculated

Our web collection on statistics for biologists contains articles on many of the points above.

Software and code

Policy information about availability of computer code

Data collection	All crystal structure data were collected at the shanghai synchrotron radiation facility (SSRF) on beamlines BL19U or BL17Uand processed with HKL2000 or HKL3000 program. ITC experiments were performed using a MicroCal iTC200 microcalorimeter system. The UHPLC-MS/MS analysis was performed on an ultra-high performance LC system (Agilent 1290 II) coupled with G6495 mass spectrometer. All the mononucleosides were separation by C18 column (Zorbax Eclipse Plus C18, 2.0 mm×100 mm i.d, 1.8 µm particle size, Agilent Technologies, Palo Alto, CA).
Data analysis	All crystal structures were built using Coot and refined by ccp4 and Phenix program.<br>The conservation of the surface of METTL4 was analyzed by ConSurf, and the diagrams of interactions involving in Am and SAM were analyzed by LigPlot. All structure pictures representing in paper were prepared with PyMOL.<br>ITC experiments were analyzed using Origin 7 software.<br>The modification level of RNA or DNA vivo and in vitro was analyzed by MassHunter Qualitative analysis B.07.00 and Graphpad Prism5.<br>For the RNA-seq, the filter reads were mapped to the Arabidopsis thaliana genome (TAIR10) by Hisat2 (version 2.2.1). High mapping quality reads were obtained using SAMtools (version 1.13). The track files which fragments were normalized as fragments per bin (bin size = 10 bp) per million mapped fragments were obtained using bamCoverage tool in deepTools (version 3.5.1) and viewed with IGV (version 2.11.2). FeatureCounts (version 2.0.1) was used to calculate the read number for each gene 50. DESeq2 (version 1.32.0) was used to identify differentially expressed genes (DEGs). Gene ontology (GO) enrichment analysis was carried out with DAVID and ClusterProfiler. Different alternatively splicing genes were identified by rMATS (version 4.1.1).

For manuscripts utilizing custom algorithms or software that are central to the research but not yet described in published literature, software must be made available to editors and reviewers. We strongly encourage code deposition in a community repository (e.g. GitHub). See the Nature Research guidelines for submitting code & software for further information.

Data

Policy information about availability of data

All manuscripts must include a data availability statement. This statement should provide the following information, where applicable:

- Accession codes, unique identifiers, or web links for publicly available datasets
- A list of figures that have associated raw data
- A description of any restrictions on data availability

Structural factors and coordinates have been deposited in the Protein Data Bank under accession codes 7CVA, 7CV7, 7CV9, 7CV8 and 7CV6 for SeMETTL4, METTL4-SAM complex, METTL4-SAH complex, METTL4-SFG complex and METTL4-Am-SAH complex, respectively.

Field-specific reporting

Please select the one below that is the best fit for your research. If you are not sure, read the appropriate sections before making your selection.

☒ Life sciences ☐ Behavioural & social sciences ☐ Ecological, evolutionary & environmental sciences

For a reference copy of the document with all sections, see nature.com/documents/nr-reporting-summary-flat.pdf

Life sciences study design

All studies must disclose on these points even when the disclosure is negative.

Sample size	No statistical methods were used to predetermine sample size for plant experiments.A minimum of 20 individual plants for flowering time were counted based on the previous publication (Bu et al., 2014) describing the sample size for rosette leaf number measurement. A detailed description of sample sizes for each experiment is given in the Figure legends or Methods section.
Data exclusions	No data was excluded from analysis in this study.
Replication	For in vivo experiments, the various modifications including RNA m6Am, m1A, m6A, Am were measured at least two independent times, and each time was repeatedly measured three times. For in vitro methylation reaction, each experiment was repeated at least two independent times, and each time was repeatedly measured three times.
Randomization	All normal growing samples were selected randomly.
Blinding	Blinding is not relevant to this study. All experiments were assigned into groups including relevant controls and analysis was done objectively and without bias.

Reporting for specific materials, systems and methods

We require information from authors about some types of materials, experimental systems and methods used in many studies. Here, indicate whether each material, system or method listed is relevant to your study. If you are not sure if a list item applies to your research, read the appropriate section before selecting a response.

Materials & experimental systems

n/a	Involved in the study
☒	☐ Antibodies
☒	☐ Eukaryotic cell lines
☒	☐ Palaeontology and archaeology
☒	☐ Animals and other organisms
☒	☐ Human research participants
☒	☐ Clinical data
☒	☐ Dual use research of concern

Methods

n/a	Involved in the study
☒	☐ ChIP-seq
☒	☐ Flow cytometry
☒	☐ MRI-based neuroimaging
